# Supplementary material for: Archaeomagnetic evidence indicates post-Inka reheating of metallurgical kilns at Quillay (NW Argentina)
Source: PLoS One. 2026 May 27;21(5):e0350160. doi: 10.1371/journal.pone.0350160 (PMC13215555; doi:10.1371/journal.pone.0350160)
Supplement: S1 Appendix — In addition, the experimental data can be found at doi.org/10.5281/zenodo.18341349 and earthref.org/MagIC/20562. (PDF) [file pone.0350160.s001.pdf]

S1 APPENDIX

This document is a companion to:
Archaeomagnetic evidence shows Inka metallurgical kilns were affected by a contemporary re-heating event (Quillay, NW Argentina).

CONTENTS

1. EXPERIMENTAL PROTOCOL
2. ROCK MAGNETISM RESULTS
3. ARCHAEOINTENSITY RESULTS
4. ARCHAEODIRECTIONAL RESULTS

The data in this study are associated with the MagIC contribution earthref.org/MagIC/20562 and can be also found at doi.org/10.5281/zenodo.18341349

More information can be provided upon request at jdelrio@ubu.es or mgomezpaccard@csic.es

1. EXPERIMENTAL PROTOCOL

Rock magnetism.

For oriented (cubic) specimens, we measured their magnetic susceptibility in a Bartington MS3 susceptibility meter, and the NRM intensity in a SQUID cryogenic magnetometer SRM 755 (2G Enterprises). NRM values are plotted against mass-normalized magnetic susceptibility (χ) to obtain Qn ratio (Koenigsberger, 1930) and evaluate whether the specimens possess a thermo-remanent magnetization. Values of Qn > 10 are commonly interpreted as indicative of thermoremanent magnetization (TRM); see below 2. ROCK MAGNETISM RESULTS for more information.

To characterise the magnetomineralogy of each colour facies in the samples (rubefied, brown, black), a small amount of material (0.200 - 0.300 g) was retrieved from each oriented field sample and ground to a fine powder in an agate mortar and pestle before being fixed in place in the VFTB's quartz holder with glass wool. The naming of VFTB specimens replicates field sample's names, making them traceable to their source material, with an added letter indicating the colour: *b* specimens belong to the black layer of the inner wall, *r* are rubefied specimens, and *br* are brown. Note that not all field samples show black layers, in which case only *r* or *br* is used (see Table C below in section 2. ROCK MAGNETISM RESULTS).

These underwent a standardised set of rock magnetism experiments: Isothermal Remanent Magnetisation acquisition curve (1T), hysteresis loop (±1T), backfield curve (-1T), and magnetization vs. temperature curve (up to 600°C and back to room temperature). The parameters derived from said experiments are the following: saturation magnetization (Ms), saturation remanent magnetization (Mrs), coercive force (Hc), remanent coercivity (Hcr), and Curie temperature (T_C), which were obtained with RockMagAnalyzer 1.1 (Leonhardt, 2006).

We reproduce in Table A the standard parameters input in the VFTB software; a copy of the .prm file used can be found in the associated repository.

Table A. Parameters used in the VFTB experiments.

**IRM:**	**Hysteresis:**	**Backfield:**	**Temperature_ramp:**
number_of_data_sets: 8<br>data_set1:<br>temperature: 0<br>dwell_time: 0<br>amplitude: 900<br>gain: 1<br>data_cycles: 13<br>wait_cycles: 10<br>field_steps: [0,0.6,1.2,1.5,2.1,3,4.5,6,7.5,9,12,15,18,24,30]	number_of_data_sets: 8<br>data_set1:<br>temperature: 0<br>dwell_time: 0<br>amplitude: 900<br>susc_amplitude: 900<br>gain: 0<br>data_cycles: 13<br>wait_cycles: 10<br>field_steps: [0,1.5,3,4.5,6,7.5,12,15,18,24,30]	number_of_data_sets: 8<br>data_set1:<br>temperature: 0<br>dwell_time: 0<br>amplitude: 900<br>gain: 0<br>data_cycles: 15<br>wait_cycles: 15<br><br>magnetization_field: 30<br>negative_field: -30<br>field_step: 0.15<br>field_limit: 3	number_of_data_sets: 2<br><br>**data_set1:**<br>temperature: 600<br>dwell_time: 10<br>amplitude: 900<br>susc_amplitude: 900<br>gain: 1<br>data_cycles: 15<br>wait_cycles: 0<br>ramp_field: 1<br>dwell_field: 1<br>ramp_slope: 25<br>pre_field: 30<br><br>**data_set2:**<br>temperature: 30<br>dwell_time: 20<br>amplitude: 900<br>susc_amplitude: 900<br>gain: 1<br>data_cycles: 15<br>wait_cycles: 0<br>ramp_field: 1<br>dwell_field: 1<br>ramp_slope: 25<br>pre_field: 0

Full-vector determination.

We determined the archaeointensity of the specimens using the Thellier-Thellier protocol (Thellier and Thellier, 1959), including partial thermo-remanence (pTRM) checks, TRM anisotropy (TRMani) tests and cooling rate (CR) corrections at the specimen level (Figure A). This workflow complies with best practices for archaeological materials (Veitch, 1984; Chauvin et al., 2000; Genevey and Gallet, 2002; Gómez-Paccard et al., 2006; see also the quality criteria on Table B below). Two collections were subjected to archaeointensity determinations independently:

1. At Géosciences-Rennes (*Ramsés* custom-made thermal demagnetiser), 45 non-oriented specimens (19 belonging to kiln QLL07, and 4 belonging to kiln QLL22) were heated up to 540-585°C in 50-30 °C steps in a 40 µT field, until their full or almost-full demagnetisation.

2. At the Paleomagnetics Laboratory at Burgos University (MMTD80 thermal demagnetiser by Magnetic Measurements), 60 oriented cubic specimens (12 per kiln) were heated up to 490-540°C in 50-30°C steps in a 45 µT field, until their full or almost-full demagnetisation.

The remanence of the specimens after each temperature step was measured in similar 2G SQUID magnetometers. We measured the magnetic susceptibility of all specimens after each increase in temperature in a Bartington MS3 susceptibility meter in order to monitor any possible magnetochemical changes due to thermal treatment. We performed partial thermo-remanence (pTRM) checks every two heating steps, which involve heating the specimens back to a lower temperature and measuring again their remanence to compare both acquisitions. For TRM anisotropy (TRMani) corrections, we performed extra steps in which the laboratory field is induced in the specimen's six orthogonal directions (±z, ±y, ±x); these steps are added when the specimen has lost 60-70% of its initial magnetisation. We used the +z phase of this extra set to calculate the cooling rate (CR) corrections by letting the specimens cool without a fan for *ca.* 25-30 hours to mimic the natural cooling process —the normal, fan-aided cooling is much quicker, about 1.5 – 3 hours). Figures SI1a and SI1b illustrate this process (the TRMani and CR steps are represented in different diagrams to facilitate comprehension).

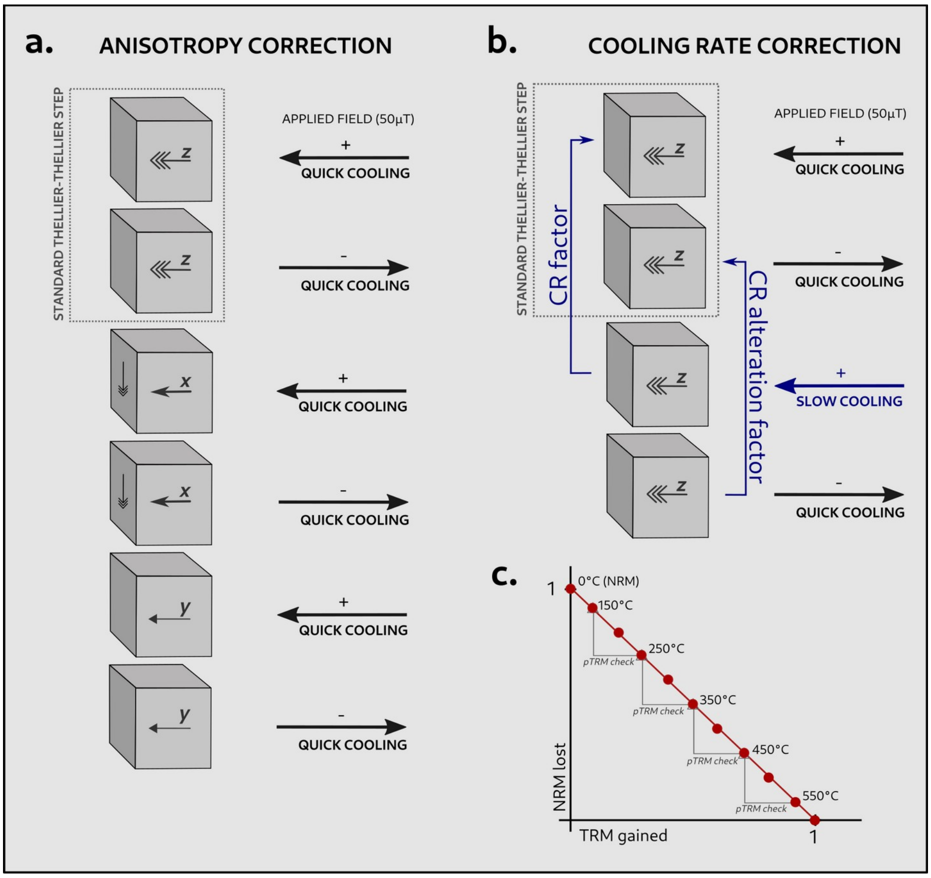

Figure A. Schematic representation of TRMani, CR, and pTRM steps in the Thellier-Thellier protocol. The figure is taken from our previous work (del Río et al., 2025).

We determined the archaeointensity both with and without TRMani and CR corrections. TRMani corrections were accepted in all selected specimens (see Table D in 3. ARCHAEOINTENSITY RESULTS for the full list). The CR correction was only applied for cases where the *CR correction* was higher than the *CR alteration factor* (Figure A-b). Where this condition is not met, we applied the averaged the correction factor from successful specimens, i.e. 0.95 (Table SI1). We excluded specimens with CR correction factors over 1.02.

The heating steps in the Thellier-Thellier protocol double as a demagnetisation sequence for directional determination. The direction of the characteristic remanent magnetisation is calculated as a best-fit line. All data analysis (intensity and direction) was done with the Starmac package, developed by Dr. Philip Roperch at Géosciences Rennes.

We accepted or rejected specimens from the initial collections based on high-standard quality criteria (Paterson et al., 2012, 2014), guided by which we consider best practices in the field of archaeo- and palaeomagnetism (e.g. Genevey and Gallet, 2002; Chauvin, Roperch and Levi, 2005; Genevey et al., 2009). These criteria can be found in Table B.

Table B. Quality criteria used in archaeointensity calculations, including the minimum or maximum acceptable value in each case. Reproduced from our previous work (del Río et al., 2025).

CRITERIA	DESCRIPTION	THRESHOLD VALUE
Linear Arai plot	Single magnetic component, no mineralogical changes due to heating	Visual assessment; related to *k*
Linear Zijderveld plot	Single magnetic component	Visual assessment
pTRM check deviation	Difference between the original temperature and the re-measured pTRM check	≤10%
Number of pTRM checks	Three successful pTRM checks in a row (Kissel & Laj, 2004)	Visual assessment
n	Number of demagnetization steps (points in the graph) involved in the calculation	≥ 6
f	Fraction of the magnetization used in the calculation of the slope (Coe et al., 1978)	≥ 0.50
k	Curvature of the slope used in calculations	0.260
β	Ratio of the standard error of the slope to the absolute value of the slope (Coe et al., 1978; Tauxe and Staudigel, 2004)	≤ 0.10
q	Quality parameter (Coe et al., 1978)	No numeric threshold, higher values denote better quality data
MAD (maximum angle of deviation	Scatter of the NRM directions (Kirschvink, 1980)	< 5º
DANG (deviation angle)	Deviation of the Zijderveld plot in relation to the origin (Tauxe & Staudigel, 2004)	< 10º

2. ROCK MAGNETISM RESULTS

Table C. Rock magnetism parameters derived from VFTB experiments. VFTB SAMPLE names that include "_1" refer to repetitions due to experiment malfunction. Bcr: remanent coercive field; S @300mT: S-parameter according to Bloemendal et al. (1992); Mrs: remanent saturation magnetisation; Ms: saturation magnetisation; Bc: coercive field; SHAPE: shape parameter according to Fabian (2003); TC1 & TC2: Curie temperatures calculated by the second derivative method. An extended version of this table can be found in the spreadsheet at https://doi.org/10.5281/zenodo.18341349.

KILN	VFTB SAMPLE	FACIES	WEIGHT (mg)	Bcr (mT)	S @300mT	Mrs (Am²/kg)	Ms (Am²/kg)	Bc (mT)	SHAPE	TC 1 (°C)	TC 2 (°C)
QLL07	Q07.05b	black	355	28.98	0.97	5.92E-02	2.90E-01	13.19	-0.79	530	--
	Q07.04r	rubefied	299	36.42	0.95	2.41E-02	1.28E-01	15.27	-0.53	552	582
	Q07.01r	rubefied	284	43.89	0.91	5.17E-03	3.61E-02	15.52	-0.56	552	--
	Q07.05r	rubefied	292	31.80	0.92	4.81E-02	1.88E-01	16.31	-0.38	545	--
	Q07.06b	black	234	28.64	0.98	1.18E-01	4.74E-01	16.39	-0.68	493	533
	Q07.08br	brown	326	31.36	0.97	6.90E-02	3.02E-01	16.8	-0.74	521	581
	Q07.02b	black	306	29.34	0.98	4.11E-02	2.37E-01	18.16	-1.23	475	--
	Q07.05br_1	brown	336	27.83	0.98	5.94E-02	1.90E-01	18.49	-0.67	565	--
	Q07.06r	rubefied	325	38.98	0.86	2.70E-02	1.51E-01	18.86	-0.72	539	--
	Q07.02r	rubefied	305	25.65	0.94	1.51E-01	3.51E-01	19.18	-0.19	542	--
	Q07.08r	rubefied	269	33.98	0.89	6.69E-02	1.97E-01	19.21	-0.02	547	--
	Q07.06br_1	brown	317	30.25	0.97	1.11E-01	3.15E-01	20.8	-0.84	561	--
	Q07.06br	brown	333	29.89	0.97	9.05E-02	3.13E-01	20.88	-0.96	551	--
	Q07.08b	black	344	34.27	0.99	9.06E-02	3.89E-01	22.28	-1.18	500	--
QLL09	Q09.07br	brown	284	23.01	0.99	8.50E-02	4.68E-01	9.73	-0.64	536	576
	Q09.09r	rubefied	297	33.01	0.95	1.91E-02	1.05E-01	11.17	-0.38	543	583
	Q09.05r	rubefied	279	30.44	0.93	9.78E-02	3.41E-01	13.24	-0.36	545	--
	Q09.06r	rubefied	293	24.61	0.92	1.58E-01	5.54E-01	14.43	-0.35	536	--
	Q09.03r	rubefied	296	33.26	0.97	1.35E-01	5.94E-01	14.46	-0.12	547	--
	Q09.07r	rubefied	290	24.14	0.80	6.13E-02	2.08E-01	15.74	0.21	536	343
	Q09.04r	rubefied	253	33.72	0.88	5.32E-02	2.18E-01	16.07	-0.15	546	--
	Q09.01r	rubefied	286	43.04	0.76	4.70E-02	1.76E-01	16.76	0.33	542	--

KILN	VFTB SAMPLE	FACIES	WEIGHT (mg)	Bcr (mT)	S @300mT	Mrs (Am^2/kg)	Ms (Am^2/kg)	Bc (mT)	SHAPE	TC 1 (ºC)	TC 2 (ºC)
	Q09.10r	rubefied	259	184.58	0.57	1.87E-02	7.29E-02	17.04	0.66	0	--
	Q09.02b	black	382	38.25	0.98	7.82E-02	3.93E-01	17.68	-0.77	496	--
	Q09.03b_1	black	339	30.13	1.00	1.56E-01	5.44E-01	17.71	-0.95	475	--
	Q09.08r	rubefied	276	38.78	0.79	6.02E-02	1.44E-01	19.32	0.57	0	--
	Q09.03br_1	brown	263	41.79	0.98	8.55E-02	2.68E-01	20.4	-0.66	547	--
	Q09.02r	rubefied	275	0.00	0.50	7.41E-02	2.13E-01	21.85	0.89	538	188
	Q09.03b	black	302	40.86	0.97	9.81E-02	3.45E-01	22.66	-0.95	548	--
	Q09.08b	black	320	40.50	0.99	2.75E-01	9.74E-01	23.36	-0.96	486	521
	Q09.01b	black	340	38.63	0.99	4.57E-01	1.29E+00	23.59	-0.81	479	--
	Q14.03r	rubefied	295	30.83	0.91	1.13E-01	4.00E-01	12.48	-0.2	551	--
	Q14.04r_1	rubefied	286	29.41	0.95	1.29E-01	5.52E-01	13.16	-0.64	548	--
	Q14.09r	rubefied	287	33.69	0.89	8.13E-03	3.57E-02	14.23	0.37	0	--
	Q14.07br	brown	204	39.07	0.94	8.02E-02	3.06E-01	14.79	-0.7	0	--
	Q14.09r_2	rubefied	249	27.34	0.92	5.26E-02	1.92E-01	15.6	-0.46	547	582
	Q14.01r_1	rubefied	238	38.06	0.95	1.46E-02	6.52E-02	15.61	-0.76	547	--
	Q14.01r	rubefied	292	39.71	0.94	1.49E-02	8.63E-02	15.61	-0.42	554	--
	Q14.08r	rubefied	288	40.55	0.92	1.21E-02	5.43E-02	15.93	-0.15	543	583
	Q14.04r	rubefied	271	34.45	0.93	1.76E-01	6.02E-01	17.06	-0.42	552	--
**QLL14**	Q14.02r	rubefied	275	28.42	0.94	1.64E-01	4.79E-01	17.42	-0.47	537	--
	Q14.06r	rubefied	313	38.10	0.99	4.59E-01	1.22E+00	24.72	-0.35	546	--
	Q14.05r	rubefied	316	53.42	0.94	1.49E-01	4.49E-01	25.06	-0.43	549	--
	Q14.01br	brown	307	41.65	0.99	3.52E-01	8.76E-01	25.23	-0.79	551	--
	Q14.06b	black	311	40.40	0.99	5.44E-01	1.37E+00	25.67	-0.74	547	--
	Q14.01b	black	297	42.65	0.99	3.41E-01	9.44E-01	26.35	-0.79	549	509
	Q14.04b	black	355	49.28	0.98	2.23E-01	6.13E-01	26.43	-0.63	363	550
	Q14.09br	brown	218	44.62	0.98	4.35E-01	1.03E+00	27.54	-0.72	544	--
	Q14.05b	black	231	47.84	0.99	3.53E-01	9.91E-01	29.64	-0.82	499	549
	Q14.08b_1	black	222	51.66	0.99	2.25E-01	6.70E-01	29.91	-0.81	498	543

KILN	VFTB SAMPLE	FACIES	WEIGHT (mg)	Bcr (mT)	S @300mT	Mrs (Am²/kg)	Ms (Am²/kg)	Bc (mT)	SHAPE	TC 1 (ºC)	TC 2 (ºC)
	Q14.04br	brown	297	54.84	0.98	1.82E-01	5.01E-01	30.59	-0.69	546	--
	Q14.07b_1	black	160	47.01	0.95	1.46E-01	4.01E-01	31.7	-0.87	--	--
	Q14.08br	brown	336	55.73	0.97	3.00E-01	8.29E-01	32.19	-0.78	551	--
	Q14.07b	black	391	56.67	0.94	4.53E-02	1.36E-01	32.78	-0.48	--	--
	Q14.07b_2	black	294	57.81	0.98	1.93E-01	4.65E-01	32.78	-0.7	549	--
	Q14.09b	black	439	65.63	0.98	4.03E-01	9.17E-01	43.51	-0.78	481	545
QLL16	Q16.01r_1	rubefied	146	62.97	0.71	2.59E-03	1.51E-02	10.16	0.89	194	574
	Q16.04r_1	rubefied	274	40.08	0.96	1.75E-02	1.09E-01	15.08	-0.57	547	--
	Q16.05r	rubefied	299	27.97	0.97	1.95E-01	6.51E-01	16.44	-0.74	553	--
	Q16.07r	rubefied	332	39.67	0.88	7.59E-02	3.51E-01	17.09	-0.76	--	--
	Q16.03r	rubefied	341	35.91	0.96	1.18E-01	4.08E-01	17.43	-0.71	551	--
	Q16.02r	rubefied	222	34.05	0.91	1.20E-01	4.44E-01	19.36	-0.85	536	--
	Q16.05br	brown	323	41.44	0.99	1.60E-01	5.20E-01	21.99	-0.69	545	--
	Q16.03br	brown	329	43.55	0.98	1.04E-01	3.11E-01	22.15	-0.69	552	--
	Q16.07br_1	brown	283.00	45.3	0.99	1.15E-01	3.43E-01	23.06	-0.75	521	--
	Q16.07br	brown	329	44.84	0.99	1.36E-01	3.99E-01	23.77	-0.68	512	--
	Q16.02br	brown	275	46.05	0.99	1.60E-01	4.25E-01	26.08	-0.75	518	--
QLL22	Q22.10br	brown	356	32.53	0.97	1.87E-02	2.40E-01	7.51	-0.56	531	581
	Q22.03r	rubefied	309	33.14	0.90	4.85E-02	3.63E-01	9.55	-0.38	517	--
	Q22.07r	rubefied	294	8.24	0.85	4.22E-02	2.23E-01	10.91	0.12	526	--
	Q22.06br	brown	281	27.51	0.99	7.62E-02	2.89E-01	11.37	-0.59	538	--
	Q22.04r_1	rubefied	290	38.07	0.97	2.91E-02	2.81E-01	11.39	-0.79	534	--
	Q22.10r	rubefied	312	33.59	0.89	1.50E-02	9.73E-02	11.44	-0.15	541	--
	Q22.08r	rubefied	284	35.33	0.94	2.62E-02	2.14E-01	12.72	-0.72	530	--
	Q22.01b_1	black	209	29.81	1.00	1.78E-01	6.07E-01	13.81	-0.85	506	--
	Q22.01r	rubefied	219	24.46	0.92	4.77E-02	1.92E-01	13.83	-0.32	554	574
	Q22.04r	rubefied	258	41.19	0.97	3.89E-02	3.08E-01	14.56	-0.95	540	--
	Q22.10b	black	275	23.41	1.00	1.53E-01	6.42E-01	14.67	-1.17	474	--

KILN	VFTB SAMPLE	FACIES	WEIGHT (mg)	Bcr (mT)	S @300mT	Mrs (Am²/kg)	Ms (Am²/kg)	Bc (mT)	SHAPE	TC 1 (ºC)	TC 2 (ºC)
	Q22.09r	rubefied	292	36.63	0.84	4.16E-02	1.72E-01	15.5	-0.05	535	--
	Q22.06r	rubefied	270	43.15	0.80	5.19E-02	2.18E-01	16.1	0.02	534	--
	Q22.08b_1	black	327	32.12	1.00	1.04E-01	3.78E-01	16.57	-0.88	490	--
	Q22.07b	black	326	29.28	1.00	1.25E-01	4.85E-01	17.27	-0.93	524	--
	Q22.08b	black	285	31.07	1.00	1.07E-01	3.91E-01	17.91	-0.93	499	--
	Q22.04br	brown	319	29.73	1.00	9.01E-02	3.19E-01	18.33	-0.6	489	--
	Q22.01br	brown	348	33.71	0.98	1.57E-01	3.83E-01	23.01	-0.62	549	--
	Q22.01b	black	263	32.22	1.00	2.10E-01	7.23E-01	26.58	-1.3	490	--

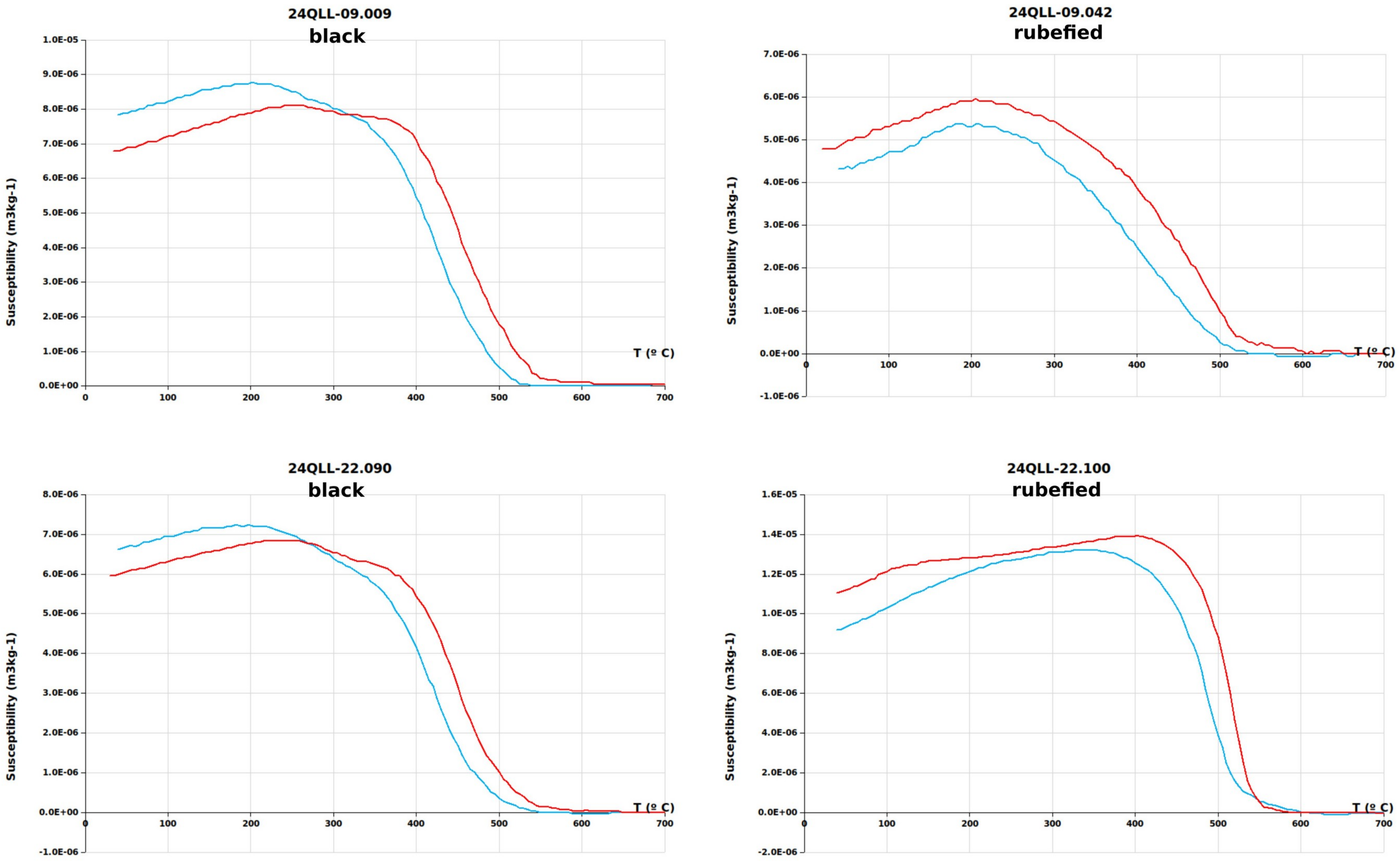

Figure B. Representative thermomagnetic curves (magnetic susceptibility vs. temperature) for two of the kilns (up: QLL09; down: QLL22).

3. ARCHAEOINTENSITY RESULTS

Table D. Intensity results at the specimen level (abridged version; the full derived parameters can be found in the accompanying spreadsheet). Spec: specimen; T1: first temperature step in intensity calculation; T2: last temperature step in intensity calculation; N: number of temperature steps included in intensity calculation; f: fraction of the TRM used in intensity calculation; g: gap factor; q: quality parameter; k: Arai plot curvature parameterMAD: maximum angle of deviation; DANG: deviation angle; β: ratio of the standard error of the slope to the absolute value of the slope; B: intensity; Tani: temperature at which the extra steps for anisotropy correction were done; fani: TRM anistropy correction factor; Bani: intensity corrected for TRMani effects; fCR: cooling rate correction factor; Bani+CR: intensity corrected for both TRMani and CR effects; k: curvature of the slope.

KILN	SPEC	T1(ºC)	T2(ºC)	N	f	g	q	k	MAD(º)	DANG(º)	β	B(μT)	Tani(ºC)	fani	Bani(μT)	fCR	Bani+CR(μT)
QLL07	07002	150	520	12	0.62	0.86	28.7	0.13	2.9	3.2	-0.02	30.3	490	1.01	30.5	0.88	26.8
	07005	150	490	10	0.9	0.88	42.7	0.09	2.4	0.4	-0.02	31.8	430	0.93	29.6	0.93	27.4
	07008	150	460	10	0.84	0.83	14.5	0.36	3.1	1.7	-0.05	31.4	430	0.86	26.9	0.88	23.7
	07027	150	520	12	0.9	0.9	66.2	0.04	2.5	1	-0.01	32.3	430	0.9	29.1	0.89	26
	07030	150	490	11	0.86	0.87	40.4	-0.13	2.8	5.1	-0.02	36.3	430	0.89	32.2	0.95	30.6
	07047	150	460	10	0.71	0.87	32.1	0.07	2.9	0.7	-0.02	28.4	430	0.96	27.2	0.92	25.1
	07063	150	520	12	0.74	0.84	44.0	0.1	1.7	3.1	-0.01	36.3	490	0.99	35.7	0.88	31.5
	07068	150	490	10	0.53	0.81	18.7	0.15	4.7	4.8	-0.02	28.3	490	1.02	28.9	0.92	26.6
	07070	150	520	10	0.85	0.85	38.2	0.06	2.9	0.6	-0.02	26.8	490	0.99	26.6	0.88	23.4
	07901C	100	490	8	0.76	0.731	18.7	-0.0736	2.7	2.6	-0.0297	32.8	400	0.969	31.8	0.95	30.2
	07901D	100	600	14	0.71	0.871	22.5	0.0825	4.9	2.3	-0.0273	29.6	400	0.868	25.7	1.091	28.0
	07902C	100	600	14	0.68	0.892	37.5	0.0273	2.6	0.4	-0.0162	28.9	400	1.014	29.3	1.087	31.8
	07903A1	100	470	7	0.928	0.747	26.0	0.1705	1.8	0.9	-0.0267	35	400	0.914	32.0	1.096	35
	07903A2	100	470	7	0.954	0.799	34.1	0.1224	1.2	0.7	-0.0223	31.3	400	1.016	31.8	0.95	30.2
	07903B2	100	440	6	0.898	0.741	43.4	-0.0742	0.9	0.1	-0.0153	28.1	400	1.018	28.6	0.95	27.2
	07903C2	100	440	6	0.917	0.753	105.7	-0.0088	1.6	0.5	-0.0065	35.6	400	0.89	31.7	0.95	30.1
	07903C3	100	400	5	0.838	0.725	24.7	0.0104	1.9	0.8	-0.0246	31.4	400	0.933	29.3	1.028	30.1
	07903C4	100	440	6	0.685	0.734	17.0	-0.1708	2.7	2.1	-0.0296	35.9	400	0.922	33.1	0.95	31.4
	07903D1	200	440	5	0.872	0.707	45.9	0.0757	2.3	0.6	-0.0134	38.6	400	0.826	31.9	0.95	30.3

KILN	SPEC	T1(ºC)	T2(ºC)	N	f	g	q	k	MAD(º)	DANG(º)	β	B(μT)	Tani(ºC)	fani	Bani(μT)	fCR	Bani+CR(μT)
	07903D2	100	440	6	0.890	0.782	53.7	0.0161	2.5	0.9	-0.0130	36.9	400	0.843	31.1	0.95	29.5
	07903D3	200	490	7	0.493	0.743	8.3	-0.0243	4.9	2.9	-0.0443	38.8	400	0.817	31.7	0.95	30.1
	079030E 1	200	440	5	0.872	0.692	41.5	0.0319	4.9	0.6	-0.0145	34.8	400	0.931	32.4	0.95	30.8
	079030E 2	200	440	5	0.791	0.718	29.6	0.0158	1.4	1.3	-0.0192	34.4	400	0.991	34.1	0.95	32.4
	079030E 3	100	400	5	0.839	0.737	23.8	0.0433	1.8	0.5	-0.0260	34.1	400	0.927	31.6	1.046	33.0
QLL09	9039	150	460	9	0.88	0.86	20.4	0	4.9	3.1	-0.04	31.5	370	0.98	31	0.88	27.3
	9080	150	520	12	0.94	0.89	44.9	0	1.9	0.9	-0.02	32.9	430	0.94	30.8	0.91	28
	9117	150	520	12	0.93	0.87	62.5	0	1.5	1.3	-0.01	33.8	490	0.99	33.6	0.88	29.6
	9147	150	520	12	0.7	0.88	40.8	0	4.7	1.5	-0.02	32	490	1.02	32.6	0.88	28.7
	9181	150	520	12	0.91	0.9	56.5	0	1.3	1.2	-0.01	32.7	370	0.92	30.2	0.82	24.8
	9252	150	520	12	0.66	0.89	30.4	0	2.1	2.2	-0.02	33.1	490	0.97	31.9	0.96	30.7
	9277	280	520	9	0.39	0.87	18.6	0	4.5	3.3	-0.02	28.9	490	0.9	25.9	0.92	23.8
QLL14	14004	150	520	12	0.89	0.88	42.9	-0.068	3.5	0.6	-0.02	35.6	490	1	35.5	0.88	31.2
	14014	310	520	8	0.77	0.83	13.6	-0.172	4.5	0.7	-0.05	37.9	490	0.98	37.2	0.88	32.7
	14022	150	520	12	0.89	0.87	76.0	0.031	4.3	1.4	-0.01	29.9	490	0.98	29.3	0.88	25.8
	14032	250	520	9	0.75	0.74	19.1	-0.044	2.2	0.7	-0.03	40	490	0.87	34.9	0.88	30.7
	14036	150	520	12	0.86	0.87	25.9	-0.145	2.6	0.7	-0.03	33	490	0.89	29.3	0.88	25.8
	14040	150	520	12	0.89	0.86	34.7	-0.087	3.2	0.2	-0.02	30.4	490	0.94	28.4	0.88	25.1
	14057	250	520	10	0.76	0.85	74.2	0.006	1.8	0.5	-0.01	38.1	490	0.96	36.7	0.88	32.3
	14073	150	520	12	0.92	0.88	92.3	-0.017	1.5	1	-0.01	34.5	490	0.96	33.2	0.94	31.3
	14103	150	520	12	0.86	0.89	58.9	0.012	2.1	0.7	-0.01	34.2	490	1	34.1	0.93	31.8
	14109	150	520	12	0.88	0.87	81.9	0.008	1.8	1.3	-0.01	34.3	490	0.97	33.2	0.91	30.3
QLL16	16003	150	430	9	0.83	0.86	58.0	0.044	1.4	1.2	-0.01	29.6	370	0.94	27.9	0.89	24.9
	16008	200	490	10	0.79	0.87	36.0	0.181	1.5	0.9	-0.02	28.5	430	0.99	28.2	0.93	26
	16015	150	520	12	0.86	0.89	51.9	0.060	2	1.1	-0.01	33.7	490	1	33.7	0.88	29.7
	16016	150	520	12	0.88	0.9	44.0	-0.049	1.9	1.3	-0.02	34.4	430	0.95	32.7	0.93	30.5

KILN	SPEC	T1(ºC)	T2(ºC)	N	f	g	q	k	MAD(º)	DANG(º)	β	B(μT)	Tani(ºC)	fani	Bani(μT)	fCR	Bani+CR(μT)
	16017	250	520	10	0.84	0.87	60.1	0.037	1.3	0.8	-0.01	32.7	490	0.99	32.4	0.96	31.1
	16023	250	520	10	0.59	0.82	33.7	0.120	2.5	1	-0.01	34.8	490	1.02	35.4	0.9	31.8
	16036	150	460	10	0.49	0.82	23.4	-0.009	4.4	6.8	-0.02	31.6	490	0.94	29.7	0.88	26.2
	16051	150	520	12	0.92	0.89	57.5	-0.034	2.9	0.4	-0.01	32.3	430	0.97	31.2	0.89	27.6
	16055	150	520	11	0.93	0.88	67.1	-0.034	4	1.1	-0.01	27.4	430	0.97	26.5	0.87	23
QLL22	22004	150	520	12	0.92	0.9	65.6	0.033	1.8	1	-0.01	32.7	430	0.9	29.5	0.91	26.7
	22007	150	460	10	0.73	0.88	46.6	0.107	1.6	0.9	-0.01	31	430	0.93	28.9	0.94	27.1
	22015	150	430	9	0.87	0.85	48.8	0.030	4.9	3.6	-0.02	33.3	370	0.92	30.8	0.88	27.1
	22019	150	460	10	0.9	0.88	36.8	0.103	2.8	1.6	-0.02	30.5	370	0.97	29.6	0.87	25.8
	22023	150	430	9	0.83	0.86	76.9	0.046	1.8	0.2	-0.01	30.5	370	0.97	29.4	0.93	27.2
	22024	150	520	11	0.91	0.9	44.6	0.093	1.6	0.7	-0.02	30.7	370	0.94	28.9	0.9	26.1
	22044	150	520	12	0.92	0.9	52.0	0.126	2.3	1.9	-0.02	29.1	370	0.97	28.2	0.89	25.2
	22079	150	430	9	0.7	0.86	22.2	0.145	3.2	0.4	-0.03	29.2	430	1.01	29.5	0.88	26
	22087	310	490	7	0.53	0.82	33.3	0.189	3	1	-0.01	29.2	370	0.96	27.9	0.91	25.3
	2201D	100	505	9	0.629	0.847	64.6	-0.0257	2.6	1.9	-0.0083	31.1	400	1.016	31.6	0.95	30.0
	2201E	100	520	10	0.636	0.876	35.0	-0.0225	3.0	0.6	-0.0159	30.9	400	0.974	30.1	1.048	31.5

4. ARCHAEODIRECTIONAL RESULTS

Table E. Directional results at the specimen level. T1 and T2: minimum and maximum temperatures for directional calculation; n 1: number of steps between T1 and T2; DEC: declination calculated through origin, without anisotropy correction; INC: inclination calculated through origin, without anisotropy correction; MAD: maximum angle of deviation calculated through origin, without anisotropy correction; DEC COR: declination calculated through origin, corrected for TRM anisotropy effect; INC COR: inclination calculated through origin, corrected for TRM anisotropy effect; MAD COR: maximum angle of deviation calculated through origin, corrected for TRM anisotropy effect.

KILN	SAMPLE	SPEC	T1(ºC)	T2(ºC)	N	DEC (º)	INC (º)	MAD (º)	DEC COR (º)	INC COR (º)	MAD COR (º)
QLL07	Q07.01	07002	150	520	12	7	-21.5	1	5.3	-21.5	1
	Q07.01	07005	150	490	10	7.7	-21.3	1.2	5.3	-23	1.3
	Q07.02	07008	150	460	10	9.1	-22.8	1.3	7.7	-27.7	1.4
	Q07.05	07027	150	520	12	10.6	-26.8	1.3	9.1	-27.6	1.4
	Q07.05	07030	150	490	11	10.5	-30.3	3.1	8.7	-35.3	3.3
	Q07.08	07047	150	460	10	13.3	-24.9	0.9	10.4	-26.6	0.9
	Q07.04	07063	150	520	12	14.2	-24.8	1	13.2	-25.3	1
	Q07.06	07068	150	490	10	3.2	-29.6	1.3	1.3	-29.5	1.3
	Q07.06	07070	150	520	10	4.9	-34.1	1.1	4	-34.6	1.1
QLL09	Q09.02	09039	150	460	9	6.1	-26.6	3.4	6.1	-29.1	3.4
	Q09.04	09080	150	520	12	11.7	-24.1	1	7.9	-25.6	1
	Q09.05	09117	150	520	12	10.2	-23.9	0.9	8.3	-24.1	0.9
	Q09.06	09147	150	520	12	13.1	-22.7	1.5	12.3	-21.8	1.5
	Q09.07	09181	150	520	12	11.9	-24.3	1	9.5	-26.7	1
	Q09.09	09252	150	520	12	13.9	-26.7	0.9	12.7	-28	0.9
	Q09.10	09277	280	520	9	22.3	-28.1	1.2	21.4	-29	1.4
QLL14	Q14.01	14004	150	520	12	10.9	-26.1	1.4	10.7	-28.1	1.4
	Q14.01	14014	310	520	8	10.8	-26.2	2	10	-28	2
	Q14.02	14022	150	520	12	12.3	-19.5	1.8	12	-20	1.8
	Q14.04	14032	250	520	9	9.6	-17.7	0.7	7.7	-27.1	0.7
	Q14.04	14036	150	520	12	8.8	-18.1	1	7.9	-25.5	1

KILN	SAMPLE	SPEC	T1(ºC)	T2(ºC)	N	DEC (º)	INC (º)	MAD (º)	DEC COR (º)	INC COR (º)	MAD COR (º)
	Q14.05	14040	150	520	12	7.2	-25.4	1.2	6.4	-26.1	1.2
	Q14.07	14057	250	520	10	8.2	-23.9	0.7	8.1	-25.3	0.7
	Q14.08	14073	150	520	12	11	-24.1	0.7	10.4	-25.3	0.7
	Q14.09	14103	150	520	12	10.2	-29.3	0.9	9.1	-33.7	0.8
	Q14.06	14109	150	520	12	8.8	-22.8	0.9	8.3	-24.4	0.9
	Q16.01	16003	150	430	9	9.2	-25	0.8	8.9	-25.4	0.8
	Q16.01	16008	200	490	10	8.7	-25.8	0.9	8.7	-27.2	0.9
	Q16.02	16015	150	520	12	8.1	-25.5	0.9	9	-26.7	0.9
	Q16.02	16016	150	520	12	7.8	-25.3	1	6.9	-27.1	1
**QLL16**	Q16.02	16017	250	520	10	7.7	-24.9	0.7	5.7	-27.1	0.7
	Q16.04	16023	250	520	10	12.1	-22.6	0.6	11.6	-23.7	0.6
	Q16.05	16036	150	460	10	4.4	-26.2	1.5	4.5	-31.4	1.5
	Q16.07	16051	150	520	12	11.8	-25	1.3	9.6	-27.3	1.3
	Q16.07	16055	150	520	11	13.2	-23.9	2	11.8	-25.6	2
	Q22.01	22004	150	520	12	15.9	-18.5	1	17.5	-20.9	1.1
	Q22.01	22007	150	460	10	14.5	-20.9	0.7	16.4	-21.5	0.7
	Q22.03	22015	150	430	9	13.3	-23.6	3.2	11.6	-23	3.5
	Q22.03	22019	150	460	10	12.6	-23.7	1.7	11.3	-24.2	1.7
**QLL22**	Q22.04	22023	150	430	9	10.8	-25.6	0.9	11.2	-25.2	0.9
	Q22.04	22024	150	520	11	9.8	-23.6	0.8	9.7	-23.8	0.9
	Q22.07	22044	150	520	12	14.1	-24.9	1.9	14.2	-24.6	1.9
	Q22.09	22079	150	430	9	10.1	-20.2	1	9.8	-19.6	1
	Q22.09	22087	310	490	7	7.3	-21.2	1.6	8.4	-21.8	1.7

Table F. Directional results at the sample and kiln level. DEC, INC, DEC COR, and INC COR as in previous table. Means (both at the sample and site level), α95, κ and standard deviation are the result of Fisher statistics; n(N): number of specimens used for calculation(number of samples to which the used specimens belong).

SAMPLE	N	DEC (º)	INC (º)	α95 (º)	DEC COR (º)	INC COR (º)	α95 COR (º)
Q07.01	2	7.4	-21.4	1.5	2.6	-22.3	11.2
Q07.02	1	9.1	-22.8	***	7.7	-27.7	***
Q07.04	1	14.2	-24.8	***	13.2	-25.3	***
Q07.05	2	10.6	-28.6	7.6	8.9	-31.5	16.9
Q07.06	2	4	-31.9	10.3	2.6	-32.1	12.2
Q07.08	1	13.3	-24.9	***	10.4	-26.6	***
**KILN**	**n(N)**	**DEC (º)**	**INC (º)**	**α95 (º)**	**DEC COR (º)**	**INC COR (º)**	**α95 COR (º)**
**QLL07**	9(6)	9.8	-25.8	4.3	7.6	-27.6	4.4

SAMPLE	N	DEC (º)	INC (º)	α95 (º)	DEC_cor (º)	INC_cor (º)	α95_cor (º)
Q09.02	1	6.1	-26.6	***	6.1	-29.1	***
Q09.04	1	11.7	-24.1	***	7.9	-25.6	***
Q09.05	1	10.2	-23.9	***	8.3	-24.1	***
Q09.06	1	13.1	-22.7	***	12.3	-21.8	***
Q09.07	1	11.9	-24.3	***	9.5	-26.7	***
Q09.09	1	13.9	-26.7	***	12.7	-28	***
Q09.10	1	22.3	-28.1	***	21.4	-29	***
**KILN**	**n(N)**	**DEC (º)**	**INC (º)**	**α95 (º)**	**DEC COR (º)**	**INC COR (º)**	**α95 COR (º)**
**QLL09**	7(7)	11.2	-24.7	2.5	9.5	-25.9	2.9

SAMPLE	N	DEC (º)	INC (º)	α95 (º)	DEC_cor (º)	INC_cor (º)	α95_cor (º)
Q14.01	2	10.9	-26.2	0.3	10.3	-28.1	1.4
Q14.02	1	12.3	-19.5	***	12	-20	***
Q14.04	2	9.2	-17.9	1.9	7.8	-26.3	3.5
Q14.05	1	7.2	-25.4	***	6.4	-26.1	***
Q14.06	1	8.8	-22.8	***	8.3	-24.4	***

Q14.07	1	8.2	-23.9	***	8.1	-25.3	***
Q14.08	1	11	-24.1	***	10.4	-25.3	***
Q14.09	1	10.2	-29.3	***	9.1	-33.7	***
**KILN**	**n(N)**	**DEC (º)**	**INC (º)**	**α95 (º)**	**DEC COR (º)**	**INC COR (º)**	**α95 COR (º)**
**QLL14**	10(8)	9.7	-22.8	2.6	9.1	-25.1	2.3

SAMPLE	N	DEC (º)	INC (º)	α95 (º)	DEC_cor (º)	INC_cor (º)	α95_cor (º)
Q16.01	2	9	-25.4	2	8.8	-26.3	3.9
Q16.02	3	7.9	-25.4	0.7	7.2	-27	2.3
Q16.04	1	12.1	-22.6	***	11.6	-23.7	***
Q16.05	1	4.4	-26.2	***	4.5	-31.4	***
Q16.07	2	12.5	-24.5	3.7	10.7	-26.5	5.7
**KILN**	**n(N)**	**DEC (º)**	**INC (º)**	**α95 (º)**	**DEC COR (º)**	**INC COR (º)**	**α95 COR (º)**
**QLL16**	7(5)	9.2	-24.8	3.1	8.6	-27	3.5

SAMPLE	N	DEC (º)	INC (º)	α95 (º)	DEC_cor (º)	INC_cor (º)	α95_cor (º)
Q22.01	2	15.2	-19.7	6	17	-21.2	2.6
Q22.03	2	13.0	-23.7	1.4	11.5	-23.6	2.7
Q22.04	2	10.3	-24.6	4.8	10.4	-24.5	4.3
Q22.07	1	14.1	-24.9	***	14.2	-24.6	***
Q22.09	2	8.7	-20.7	6.1	9.1	-20.7	5.6
**KILN**	**n(N)**	**DEC (º)**	**INC (º)**	**α95 (º)**	**DEC COR (º)**	**INC COR (º)**	**α95 COR (º)**
**QLL22**	9(5)	12.3	-22.7	3.3	12.4	-22.9	3.3

REFERENCES.

Chauvin, Garcia, Lanos, and Laubenheimer. “Paleointensity of the Geomagnetic Field Recovered on Archaeomagnetic Sites from France.” *Physics of the Earth and Planetary Interiors* 120, no. 1 (2000): 111–36. https://doi.org/10.1016/S0031-9201(00)00148-5.

Chauvin, Roperch, and Levi. “Reliability of Geomagnetic Paleointensity Data: The Effects of the NRM Fraction and Concave-up Behavior on Paleointensity Determinations by the Thellier Method.” *Physics of the Earth and Planetary Interiors* 150, no. 4 (2005): 265–86. https://doi.org/10.1016/j.pepi.2004.11.008.

del Río, Cruz, Gómez-Paccard, et al. “The First Archaeomagnetic Age at Tiwanaku and Implications for Dating Andean Metallurgical Furnaces.” *Archaeometry* n/a, no. n/a (2025). https://doi.org/10.1111/arcm.70046.

Fabian (2003). Some additional parameters to estimate domain state from isothermal magnetization measurements. *Earth and Planetary Science Letters*, *213*(3-4), 337-345. https://doi-org.ezp.sub.su.se/10.1016/S0012-821X(03)00329-7

Genevey, Gallet, Rosen, and Le Goff. “Evidence for Rapid Geomagnetic Field Intensity Variations in Western Europe over the Past 800 Years from New French Archeointensity Data.” *Earth and Planetary Science Letters* 284, no. 1 (2009): 132–43. https://doi.org/10.1016/j.epsl.2009.04.024.

Genevey and Gallet. “Intensity of the Geomagnetic Field in Western Europe over the Past 2000 Years: New Data from Ancient French Pottery.” *Journal of Geophysical Research: Solid Earth* 107, no. B11 (2002): EPM 1-1-EPM 1-18. https://doi.org/10.1029/2001JB000701.

Gómez-Paccard, Chauvin, Lanos, Thiriot, and Jiménez-Castillo. “Archeomagnetic Study of Seven Contemporaneous Kilns from Murcia (Spain).” *Physics of the Earth and Planetary Interiors* 157, no. 1 (2006): 16–32. https://doi.org/10.1016/j.pepi.2006.03.001.

Koenigsberger. “Größenverhältnis von Remanentem Zu Induziertem Magnetismus in Gesteinen; Größe Und Richtung Des Remanenten Magnetismus.” *Zeitschrift Für Geophysik* 6 (1930): 190–207. https://doi.org/10.23689/fidgeo-3212.

Leonhardt, R. (2006). Analyzing rock magnetic measurements: The RockMagAnalyzer 1.0 software. *Computers & Geosciences*, *32*(9), 1420-1431. https://doi-org.ezp.sub.su.se/10.1016/j.cageo.2006.01.006

Paterson, Heslop and Muxworthy. “Deriving Confidence in Paleointensity Estimates.” *Geochemistry, Geophysics, Geosystems* 11, no. 7 (2010). https://doi.org/10.1029/2010GC003071.

Paterson, Biggin, Yamamoto, and Pan. "Towards the Robust Selection of Thellier-Type Paleointensity Data: The Influence of Experimental Noise." *Geochemistry, Geophysics, Geosystems* 13, no. 5 (2012). https://doi.org/10.1029/2012GC004046.

Thellier and Thellier. "Sur l'intensite Du Shamp Magnetique Terrestre Dans Le Passe Historique et Geologique." *Ann. Geophys.* 15 (1959): 285–376. https://cir.nii.ac.jp/crid/1573105974836722944.

Veitch. "An Investigation of the Intensity of the Geomagnetic Field during Roman Times Using Magnetically Anisotropic Bricks and Tiles." *Arch. Sci. Geneve.* 37, no. 3 (1984): 359–73. https://cir.nii.ac.jp/crid/1570572701697925632.
