# Supplementary material for: Archaeomagnetic evidence indicates post-Inka reheating of metallurgical kilns at Quillay (NW Argentina)
Source: PLoS One. 2026 May 27;21(5):e0350160. doi: 10.1371/journal.pone.0350160 (PMC13215555; doi:10.1371/journal.pone.0350160)
Supplement: Attachment — Submitted filename: Response to Reviewers.pdf [file pone.0350160.s003.pdf]

Burgos, February 26, 2026

GENERAL COMMENTS

All three reviewers, plus the editor, noticed that the title of our previous manuscript conceded preferential value of one of the hypothesis discussed in the text. We have since changed the title in order to make it less leading, just pointing out that an episode of re-heating was detected through archaeomagnetism.

We offer our answers in a different colour for ease of reading. Please note that the line numbers and mentions to tables and figures below refer to those in the reviewed manuscript without tracked changes. A file with changes tracked has also been uploaded.

ANSWER TO THE EDITOR

Dear Dr. Mroczek,

Thanks for your detailed response and suggestions to our manuscript. We provide below answers to the numbered journal requirements:

1. When submitting your revision, we need you to address these additional requirements. Please ensure that your manuscript meets PLOS ONE's style requirements, including those for file naming.

> We have changed the title, included the required short title, and changed the affiliations to comply with PLOS ONE standards.

2. Please include a complete copy of PLOS' questionnaire on inclusivity in global research in your revised manuscript. Please upload a completed version of your questionnaire as Supporting Information when you resubmit your manuscript.

> We have now submitted the inclusivity questionnaire as supplementary information.

3. In your manuscript, please provide additional information regarding the specimens used in your study. Ensure that you have reported human remain specimen numbers and complete repository information, including museum name and geographic location. (...) If no permits were required, please include the following statement: 'No permits were required for the described study, which complied with all relevant regulations.'

> Our study does not include human remains and complies with archaeological regulations in Argentina; we have expressed it as such in lines 174-175 of the revised manuscript.

4. We note that the grant information you provided in the 'Funding Information' and 'Financial Disclosure' sections do not match. When you resubmit, please ensure that you provide the correct grant numbers for the awards you received for your study in the 'Funding Information' section.

We have fixed the inconsistencies between the FUNDING INFORMATION and the FINANCIAL DISCLOSURE sections.

5. Please state what role the funders took in the study. If the funders had no role, please state: "The funders had no role in study design, data collection and analysis, decision to publish, or preparation of the manuscript."

We have added the role of the funders to our FUNDING INFORMATION section.

6. Thank you for uploading your study's underlying data set. Unfortunately, the repository you have noted in your Data Availability statement does not qualify as an acceptable data repository according to PLOS's standards. At this time, please upload the minimal data set necessary to replicate your study's findings to a stable, public repository (such as figshare or Dryad) and provide us with the relevant URLs, DOIs, or accession numbers that may be used to access these data.

Our dataset is now uploaded to an open repository (Zenodo), as well as to the MagIC palaeomagnetic database. We have added a DATA AVAILABILITY section at the end of the manuscript.

7. When completing the data availability statement of the submission form, you indicated that you will make your data available on acceptance. We strongly recommend all authors decide on a data sharing plan before acceptance, as the process can be lengthy and hold up publication timelines.

Due to the nature of the MagIC database, datasets that are associated with a publication cannot be made public until a DOI for said publication is provided. While the dataset is already uploaded, we can only validate it when we assign a final DOI to it. In the meantime, we have also made available our data at Zenodo (see previous point).

8. We note that Figure 1 in your submission contain satellite images which may be copyrighted. All PLOS content is published under the Creative Commons Attribution License (CC BY 4.0), which means that the manuscript, images, and Supporting Information files will be freely available online, and any third party is permitted to access, download, copy, distribute, and use these materials in any way, even commercially, with proper attribution. (...) We require you to either present written permission from the copyright holder to publish these figures specifically under the CC BY 4.0 license, or remove the figures from your submission.

Figure 1 was redone from public data available from the National Argentinian Geographic Institute (IGN de la República Argentina) and the NASA ASTER Digital Elevation Model V004. As these products have lesser spatial resolution (30m) than

our previous option, we decreased the zoom over the interest area, which in turn allows for a more complete version of the surrounding landscape.

9. We note that Figures 2 and SI1 in your submission contain copyrighted images. All PLOS content is published under the Creative Commons Attribution License (CC BY 4.0), which means that the manuscript, images, and Supporting Information files will be freely available online, and any third party is permitted to access, download, copy, distribute, and use these materials in any way, even commercially, with proper attribution. (...) We require you to either present written permission from the copyright holder to publish these figures specifically under the CC BY 4.0 license, or remove the figures from your submission.

We are the holders of the copyright of Figure 2 in the main manuscript, as it is an original creation. We are also the copyright holders of the old Figure SI1, which is now Figure 7 in the main manuscript. We have stated so in the figure captions.

10. Please upload a new copy of Figures 1 – 6, as the detail is not clear.

Figures 1-7 have been reuploaded with higher quality (600dpi).

11. Please include captions for your Supporting Information files at the end of your manuscript, and update any in-text citations to match accordingly.

We have re-named the supplementary information as S1 Appendix, and updated all references within the main manuscript correspondingly.

12. If the reviewer comments include a recommendation to cite specific previously published works, please review and evaluate these publications to determine whether they are relevant and should be cited. There is no requirement to cite these works unless the editor has indicated otherwise.

All the suggested citations by the reviewers were appropriate, and have been adopted.

Regarding your further comments, we also provide point-by-point answers:

The CURRENT TITLE does not accurately reflect the evidential scope of the manuscript. It presents a specific interpretative explanation-intentional slag re-smelting in modern times-as an established result, whereas the study itself demonstrates only the occurrence of a late reheating event recorded archaeomagnetically in the upper parts of the furnaces. The causal mechanism responsible for this reheating is inferred and discussed, but not independently demonstrated, and alternative explanations are explicitly considered in the manuscript. The title therefore overstates the degree of interpretative certainty supported by the data. The authors are required to revise the title so that it reflects the documented observation and dating of a late reheating event, rather than asserting a specific technological practice as a confirmed outcome.

We have changed the title of our manuscript to better reflect the empirical results; i.e. we have left out the possible causes of the re-magnetisation, pointing only to the fact that we could ascertain a re-firing event.

The current set of KEYWORDS largely duplicates terms already used in the title and therefore adds limited descriptive or indexing value. The keywords should be revised to avoid repetition and to better reflect the core problem addressed in the manuscript, rather than restating the general methodological or regional context.

We have changed the keywords to make them less reiterative.

The ABSTRACT overstates the study's conclusions by presenting intentional slag re-smelting as a confirmed result, whereas the data demonstrate only a late reheating event recorded archaeomagnetically. The interpretative nature of the proposed mechanism and the existence of alternative explanations are not adequately reflected. The abstract must be revised to align its claims with the evidential limits of the study.

We have also modified the abstract according to your suggestions, focusing on the re-heating event rather than its possible causes. It now matches the content of the manuscript.

The overall STRUCTURE OF THE MANUSCRIPT requires revision. At present, empirical results are intermixed with interpretative elements, particularly in sections dealing with comparisons to geomagnetic field models and archaeomagnetic dating. These parts go beyond the presentation of measured data and belong analytically to interpretation rather than to the Results section.

The manuscript should be REORGANISED so that the Results section is limited to the presentation of empirical data and their statistical treatment, while archaeomagnetic dating and model-based comparisons are clearly separated and placed in an appropriate analytical or discussion section. In its current form, the structure obscures the boundary between results and interpretation and encourages overstatement of the study's conclusions.

Attending to your suggestions, we have re-structured the manuscript. It now includes more sub-sections which we believe will provide a better reading experience and will make the argument more prominent. These modified sections include a separate heading for C14 dating in which we further discuss the datings, and separate subsections within the DISCUSSION that analyse separately the geomagnetic values recorded by the kilns, the archaeomagnetic dating and its comparison with available C14 dates, the hypotheses for the remagnetisation, and the limitations of our study. Notably, we have removed all interpretative paragraphs from the results section and moved them to the DISCUSSION.

The CONCLUSIONS section goes beyond what is securely demonstrated by the data. While the archaeomagnetic results clearly indicate a late reheating event affecting the upper parts of the furnaces, the conclusions present intentional slag re-smelting as the most likely explanation, rather than as a working hypothesis. The limitations of sampling and the unresolved nature of the causal mechanism are not sufficiently reflected. The Conclusions should be revised to align strictly with the empirical findings, to state uncertainties explicitly, and to avoid privileging a single interpretative scenario where the evidence remains indirect.

> We have modified the Concluding remarks, which now present both hypotheses as equally plausible and state the need of further work in order to fully understand the processes that re-magnetised the kilns.

ALL FIGURES use a grey background, which reduces clarity and is not appropriate for publication. The background should be removed and all figures should be prepared on a clean white background to improve readability and ensure consistency with journal standards.

> The figures have been remade according to the standards (white background, higher dpi).

The SUPPORTING INFORMATION requires substantial revision. At present, it contains several issues that undermine clarity and confidence in the presentation of the data. Large specimen-level tables include values that are difficult to interpret without additional guidance, including very large $\alpha95$ confidence parameters and results based on extremely low numbers of specimens, which should either be explicitly justified or excluded from summary statistics. Correction factors for anisotropy and cooling rate vary widely, in some cases reaching unusually high values, yet these are not consistently explained or contextualised. Moreover, it is not always clear which data were retained for the final calculations and which are provided solely for completeness. The Supporting Information also mixes raw data, derived results and interpretative material without a clear structure. Finally, Figure SI1 is directly relevant to the interpretation of surface processes and sampling strategy and would be more appropriate in the main text. The Supporting Information should be carefully checked for numerical accuracy and internal consistency, streamlined, and reorganised so that it clearly supports the main manuscript rather than functioning as an unfiltered data dump.

> We have reorganised the supplementary information, now called S1 Appendix, following both your suggestions and those of other reviewers. It now includes a detailed experimental protocol, the full tables for rock-magnetic, intensity, and directional parameters (all of which have been revised and are final, i.e. all values shown in the tables were included in the calculations), and new figures for ancillary experiments. The old figure SI1 is now on the main manuscript as Figure 7.

FIGURE 1 requires revision in both its caption and cartographic design. The figure clearly consists of two panels, combining a regional location map of South America with a local-

scale image of the Quillay site, yet the current caption treats it as a single satellite image and does not distinguish between these different spatial scales. In addition, the use of an orthophotomap as the main local representation is of limited analytical value. While visually informative, it does not adequately convey the geomorphological and topographic context that is relevant to the interpretation of surface exposure, site setting and potential post-depositional processes. A simplified topographic map, a DEM-based representation, or preferably a geomorphological map would better support the interpretative aims of the study. The authors should revise Figure 1 accordingly, either by replacing the orthophotomap or by complementing it with a more informative geomorphological or topographic representation.

> Figure 1 was redone from public data available from the National Argentinian Geographic Institute (IGN de la República Argentina) and the NASA ASTER Digital Elevation Model V004. As these products have lesser spatial resolution (30m) than our previous option, we decreased the zoom over the interest area, which in turn allows for a more complete version of the surrounding landscape.

TABLE 1. First, the table mixes dates obtained specifically for this study with dates taken from the literature, yet the implications of this heterogeneity are not discussed, and the referenced sources are not clearly cross-linked at the table level. Second, the treatment of multiple calibrated probability ranges (eg. for QLL09) is not sufficiently explained, and it is unclear how these alternative solutions are handled in the chronological interpretation. Third, the attribution of the QLL07 date to an old-wood effect is discussed in the text but not clearly flagged in the table itself, which may mislead readers treating the radiocarbon ages at face value. The table would benefit from clearer annotation indicating which dates are considered representative of kiln use and which are treated as problematic or interpretatively discounted.

> Attending to your and other reviewer's suggestions, Table 1 has been remade and its implications explained within the main text. We added the references for older datings in order to prevent confusion with the dates obtained *ex profeso* for this study. As you point, we do consider the age available for kiln QLL07 as a produce of the old wood effect; we still keep it as any other one in Table 1, since Fig 6 later utilises this dating as-is.

TABLE 2. The rationale for treating uncorrected directions as final while applying anisotropy and cooling-rate corrections to intensity data is not explicitly explained in the table or its caption. In addition, the meaning of the nD/nD and nI/nI ratios is not immediately clear and should be defined more transparently. The table does not indicate whether any specimens were excluded from the final means or how specimen-level variability was propagated to kiln-level results. While Table 2 is acceptable in principle, these issues should be addressed to ensure full transparency and consistency with the methodological description and Supporting Information.

> Table 2 has been changed, and we now consider both intensity and directional values corrected for TRM anisotropy and cooling rate effects. We clarified in the

legend the meaning of “nd(ND)” and “ni(NI)”, i.e. the number of specimens per sample for directional and intensity determination, respectively. We would also like to note that all the results reported in our study correspond to the specimens that were kept, and that the rejected specimens do not appear in Table 2 in the main manuscript, nor in Tables D and E in the S1 Appendix. The full raw data, including rejected specimens, are, however, available in the open repository.

The presentation of TABLE 3 is problematic and requires revision. Although the table header indicates three global geomagnetic models, the structure of the table is ambiguous, as multiple age intervals are listed within individual cells without a clear explanation of their origin or status. It is not evident which intervals correspond to primary solutions and which represent alternative or secondary model outputs. In particular, early Common Era age ranges (e.g. 0-150 CE) are presented alongside late nineteenth–twentieth century ranges without clarification of their statistical relevance or interpretative weight. Moreover, the table does not specify whether the reported age ranges are derived from directional data, intensity data, or their combination, nor does it indicate how multimodal probability solutions were treated. As a result, the table gives the impression that all age intervals are equally valid, despite their clear inconsistency with the archaeological context. This lack of hierarchy and explanation significantly reduces the clarity and interpretability of the results. Table 3 should be restructured or expanded with explicit clarification of the modelling approach, the nature of multimodal solutions, and the criteria used to identify the age ranges discussed in the text. In its current form, the table does not meet the standards of transparent and unambiguous presentation of archaeomagnetic dating results.

Table 3 has been subsequently modified. Note that there are no “secondary” results in archaeomagnetic dating and that all the listed ranges have the same probability with regards to a GMF value. This includes dates that may appear as inconsistent, such as those that pointed to the beginnings of the common era —it is archaeological context which determines the most plausible date—. Nevertheless, we performed anew the archaeological dating, since now we take the corrected directional and intensity values as final and this varied slightly the results.

In several places, archaeomagnetic dating is treated not only as a method for constraining the timing of a reheating event, but also as evidence for its causal mechanism. This distinction should be made clearer, as archaeomagnetic data date heating but do not, by themselves, identify whether the process was anthropogenic or natural.

We have now included a separate section in which we assess the archaeomagnetic dating results; you can find it in the Discussion (line 392 and onwards). We hope that this change clearly solves the issue that you rightly raised, in which you pointed that archaeomagnetic dating does not have explanation potential about the causes of the re-heating event. The discussion now includes clear separations between the dating, the hypotheses, and the limitations, and acknowledges that the current study cannot provide a definite answer to what the cause of the re-magnetisation was.

The pronounced mismatch between the radiocarbon chronology and the archaeomagnetic ages is described but not sufficiently analysed as a methodological and interpretative issue. A more explicit discussion is needed to explain why the radiocarbon dates consistently align with the archaeological context while archaeomagnetism records a much later episode.

> As you and other reviewer suggested, we have included an explicit discussion of the mismatch between archaeomagnetic and radiocarbon datings. We attribute this discrepancy to the C14 dating the Inka archaeological layers, and archaeomagnetism dating the last heating of the structure; we have provided the explanation as such in lines 441-446 in the DISCUSION and lines 605-608 in the CONCLUDING REMARKS.

The sampling strategy is limited to the upper parts of the furnaces, yet conclusions are drawn about the reuse and function of the structures as a whole. This limitation should be more clearly reflected in the scope and strength of the interpretative claims.

> The DISCUSSION now includes a full sub-section detailing the limitations of the study, namely the sampling of only the upper chambers, and the reasons why. We also provide future lines of work that will help us decipher the causes of the reheating, and thus of the remagnetisation.

Alternative natural heating processes, such as surface fires, are mentioned but not explored in a fully balanced way. It should be clarified which aspects of the archaeomagnetic signal cannot be readily explained by natural processes and which remain ambiguous.

> Since now both hypotheses (the wildfire origin, and the intentional re-use origin) are presented in a balanced way, we believe that the causes of the recent remagnetisation of the kilns are accounted for. In other words, there are no aspects of the contemporary remagnetisation that cannot be explained by natural processes, as both possibilities are acknowledged as equally explanatory.

Intensities are reported after anisotropy and cooling-rate corrections, whereas directional data are treated as final without equivalent correction. Even if methodologically justified, the rationale for this approach should be stated more clearly to ensure internal consistency.

> As mentioned earlier, we have changed our approach to the selection of final data, and have accepted the TRM anisotropy and cooling rate corrections for both intensity and directions.

Throughout the manuscript, the distinction between empirical results, interpretative inferences, and working hypotheses is not always sufficiently clear, particularly with respect to the proposed reuse scenario, which at times appears more certain than the available evidence allows.

> You highlighted how the distinction between experimental results, their implication, and our tentative hypotheses was not sufficiently clear. We modified slightly our

Results section, including in it only the presentation of the obtained data and deleting any mention to its possible interpretation. We have moved all interpretation-like statements to the Discussion section. We hope that the re-structuring of the paper and the sub-division of the Discussion in explicit headers contributes to clarify this issue.

ANSWER TO REVIEWER 1

Dear reviewer,

Thanks for the attentive reading of our manuscript. We will provide detailed answers to your comments below, together with line numbers where you can find the effected changes:

There are two hypotheses presented: 1) the site was reused (Reuse hypothesis) in the modern period or 2) the data are the result of wildfires (Wildfire hypothesis). The title of the paper betrays the authors' preferred hypothesis--that this is a modern reuse of the ancient technology. However, the authors also clearly state that that the archaeomagnetic data could have been produced from wildfires. It seems more effective to present both hypotheses as feasible upfront and then outline the data that supports/rejects each of these. It might be a better strategy than to put the Wildfire hypothesis in the Discussion. (...) I would take out "slag re-smelting in modern times."

> Accepting the suggestions of all reviewers and the editor, we have changed the title, which we believe now reflects more generally the empirical evidence of a re-heating episode without pointing to an origin hypothesis.

The authors state that this kiln reuse is a "practice documented at other Andean metallurgical sites". Likewise, they state that "historical records of metal recycling in the region during the late 19th and early 20th centuries documented in other Andean metallurgical sites" supports their favored hypothesis. This would be strong evidence for the Reuse hypothesis. However, I can find no description of such sites in their literature review. Therefore, they must include brief descriptions and references to these historical records.

> You point to the absence of other examples of kiln re-firing (during Colonial and contemporary times) in our literature review. We have chosen to provide such examples in the Discussion instead, as we believe it contributes more organically to our argument development. There, we discuss and contextualise modern practices of *pirquinería*, and provide historical sources for the reuse of old metallurgical kilns.

The manuscript mentions: "small-scale metallurgical operations carried out by marginal or impoverished individual workers (56,61,62)". The titles of the two of the three references do not seem to cover the modern period. Reference 62 lists "1st to 15th centuries". I did a brief read of this outstanding article, but I could not find a reference to the modern reuse of pre-Columbian sites. I may have missed something. Ref. 56 is about fire management. Perhaps a relevant quote from ref. 56 would be useful. I could not access the manuscript. It should be reproduced in Supplementary materials. Ref 61 seems appropriate but some brief description of the sites would be warranted. I did a quick read and could not find any references to the reuse of kilns from pre-Colonial archaeological sites, though I could have missed something. The manuscript mentions Lechtman in Ref. 59 but the article is by another author (should this be ref. 60?). The provided link to this article did not open but I

was able to get it directly. Again, my superficial read did not find any reference to the reuse of ancient sites, but I may have missed it.

There was a clear mismatch between the in-text references and the reference list that we did not notice. Thanks for your patient observation of this. The reference to Lechtman (1976) has since been fixed; in addition, we have reviewed and updated all references and their links.

While it is true that the paper by Ratto et al. (2019), "Initial study of metallurgical technology from western Tinogasta, Catamarca, NW Argentina (1st-15th centuries CE)" focuses on centuries 1st to 15th, pirquineo is mentioned in page 65 as a contemporary practice, starting in the region at the beginning of the 20th century and explained as follows:

> "The work was carried out *a pirquén*, a type of mine leasing involving cash payment or a share of production. This system coexisted with the more traditional *pirquén* industry that was carried out by a miner or group with little capital investment or technology (Godoy Orellana, 2016)".

We have since included more references that sustain the possibility of a re-use at Quillay; please find them in lines 491-512.

The article by Lechtman (Ref. 60) does list several metallurgy sites with modern, Colonial and pre-Colonial occupations. These could be excellent candidates to support the modern reuse hypothesis. To be convincing, the authors should discuss two or more of these sites showing how Quillay fits into a broader pattern. This would strongly support the Reuse hypothesis and Quillay.

We have since included more references and direct quotations that sustain the possibility of a re-use at Quillay; please find them in lines 491-512.

A major observation against the Reuse hypothesis is: why are there no 19th or early 20th century artifacts at Quillay similar to those that dated the site as Inka? One would expect at least a few artifacts from the modern period if people worked the kilns. The photographs in ref. 57 of the Pirquineros shows plenty of material artifacts and other refuse surrounding their work sites. This absence must be explained. Also, why are there no carbon dates that came back as modern? Perhaps these were not sampled. If so, simply state this. Finally, the Reuse hypothesis would be supported if there was an historical settlement nearby. GoogleEarth shows no settlements and the manuscript is silent on this issue.

You mentioned the absence of early 20th century materiality at the site and the nonexistence of historical settlements at or around Quillay as arguments against the re-use hypothesis. The absence of contemporary material remains does not strike us as too surprising: the tools used in *pirquineo* and mining activities are re-usable and valuable, and as such it seems the most logical strategy to bring them from site to site instead of leaving them behind; this explains, in our view, the fact that no tools are found. Regarding the existence of settlements, there is, indeed, a small inhabited dwelling area in Quillay itself, West of kilns QLL07, QLL09, QLL14 and

QLL16. However, the people there make a living as cattle breeders, not as miners (see the satellite image below), as they themselves told us during our fieldwork.

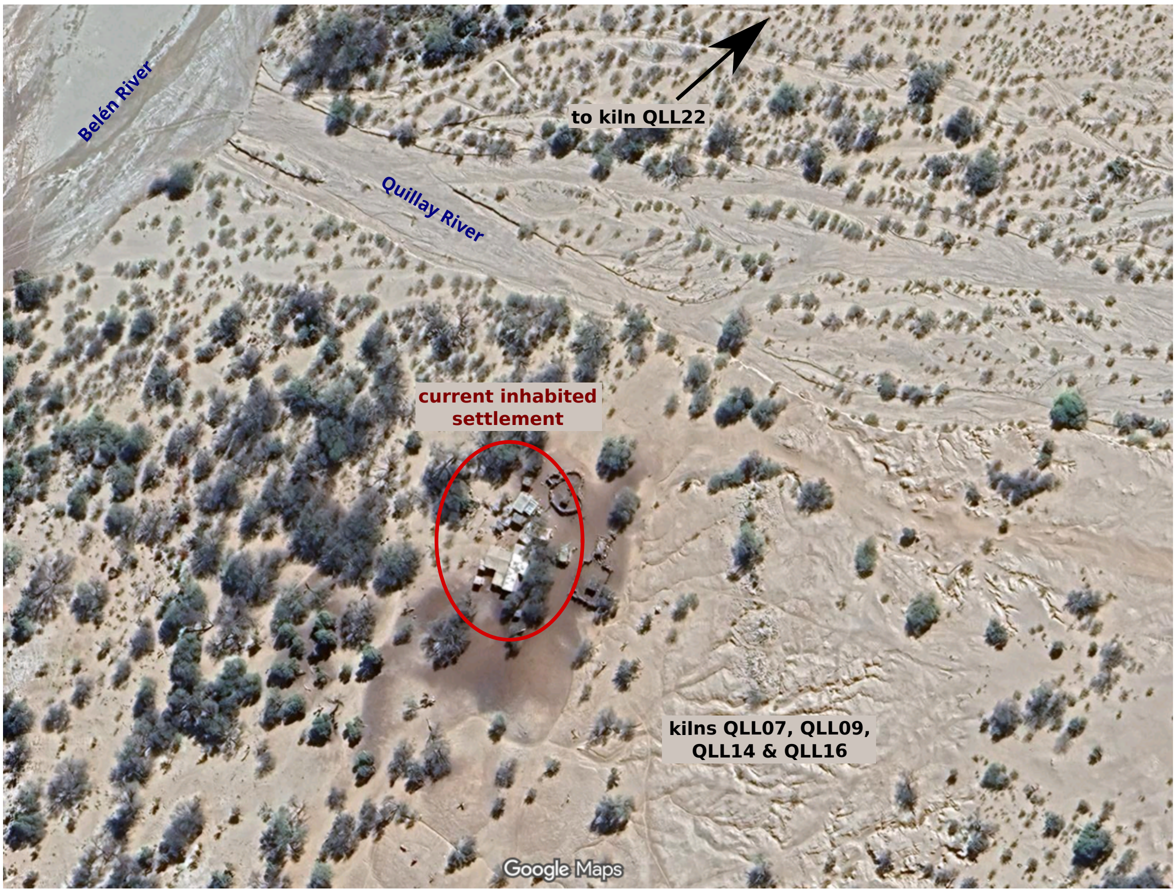

We must emphasise that *pirquinería* activities are not associated with settlements near the sources of metal. Instead, pirquineros travel from source to source, extract the materials, and get back on the route, not establishing any permanent camp. This would also account for the lack of visible remains —although traces of human activity may remain, of which the identified re-heating event could be one.

The lack of dating for the modern charcoal accumulations (now in Figure 7 in the main manuscript) is now explicitly addressed in the DISCUSSION, where we consider further work.

Dates should be expressed in calibrated formats, e.g. 1400 calAD or calCE. Ln 85: “infantile” should be “infant”. Ln. 147: “coherent’ should be “consistent”

We implemented all the changes related to orthography and formatting.

Ln 133: “Samples marked with an asterisk were dated specifically for this study at the CNA; the rest were retrieved from the available literature.” The “rest” of these dates must be specifically cited with full reporting format.

Table 1: Carbon dates should include the δ13c along with the nature of the material sampled--wood, seeds, carbon flecks etc. Some labs do not report δ13c in which case you simply write “N/A”.

The date for kiln QLL07 is dismissed as too old. However, the 14c date provided overlaps (barely to be sure) the 2-sigma of three other dates and cannot be considered as an outlier. The old wood factor is certainly a possibility. In this case, it would be good to know what the material was that they dated. The δ13c could clarify this.

> We added the references to the previously published C14 datings for our kilns, both in the caption for Table 1 and in the table itself, which we have modified according to your suggestions to include δ13C values. You can now see these changes in Table 1 in the main manuscript. In addition, there is now a brief discussion about the ranges of the available C14 dates (lines 147-161).

Ln. 191 "Our data selection approach is consistent with recent high-quality archaeointensity and archaeodirectional studies (e.g. 38,39)." Refs. 38 and 39 include the corresponding author of the present manuscript as a co-author. It would be more effective to cite an independent international standard or another independent lab.

> As you suggested, we changed the references to the studies we consider best-practice in archaeomagnetism in order to incorporate studies by other laboratories and researchers. The changes are in line 218.

Fig. SI1 is very important and should be in the main text in my opinion.

> We included the old Figure SI1 in the main manuscript; it is now Figure 7 in the text.

Data to support the Reuse hypothesis include:
Confirmation that there was an earlier reuse. In this case it is the Inca and this is confirmed / Historical data showing that reuse occurred in other similar contexts. None is presented. / Archaeological remains from the time of the supposed reuse. None is presented. / Archaeometric (14c or archaeomagtic). No 14c but archaeomag is presented. Settlement data demonstrating that there was an early Modern period settlement. No data are presented.

Data to support the wildfire hypothesis:
Historical data showing that wildfires occurred. Historical data are presented. / Lack of archaeological data since wildfires are natural. Confirmed. / Lack of a Modern settlement on or near the kilns. Unclear.

In short, assuming that the field and lab methods were not faulty, the Wildfire hypothesis is supported, the Reuse hypothesis is not.

> You provided a great summary of the arguments in favour of each of our proposed hypotheses. We have re-structured and expanded the discussion, including more data from archaeological and ethnographical observations previously published that provide further foundation for the re-use hypothesis. Please find these explanations under the heading "Exploring the causes of contemporary re-magnetisation" in the DISCUSSION (line 447 onwards). We have now added more in-text citations with ethnographic and archaeological data that substantiate the re-use hypothesis. It is our belief that the discussion is now more balanced and nuanced. We also explicitly acknowledge the open-endedness of this work, insofar as it cannot provide definite

answers. You can now find a section titled “Limitations of the study and further work”, from line 532 onwards.

ANSWER TO REVIEWER 2

Dear Dr. Kosterov,

Our most sincere thanks for your careful review of our manuscript —specially in what concerns the magnetic mineralogy of our samples, which we found very useful. We have provided below answers to each of the points you raised.

1) Experimental setup.
It would be helpful if the authors include the detailed description of experimental setup in the Supporting information, in addition to referring a reader to previous studies.

In the description of Thellier experiments, list the normal cooling time. How exactly was the CR correction calculated? Did you acquire a total TRM by cooling for 24 hours and then compare it with TRM acquired during a 'normal' cooling?

> We have included detailed laboratory protocols in the SI, with more information about the "normal" quick cooling times and the "slow", natural-like, cooling times. You can now find that information at the beginning of the supplementary information S1 Appendix.

For the anisotropy correction, list temperature(s) at which the additional pTRMs in ±X, Y, Z directions were acquired for each specimen. This information can be conveniently included in the Table SI1.

> We added the specific temperatures at which the TRM anisotropy correction steps were performed; you can now see those changes in Table D in S1 Appendix.

For hysteresis measurements, detail how the data were corrected for the high-field slope. Note that the samples like the one shown in Fig. 3d do not reach magnetic saturation in the 1 T field, and therefore the Ms value would be underestimated, perhaps significantly. Please check the field used to measure thermomagnetic curves (lines 199-200). 30 mT seems to be a typo; curves shown in Fig. 3 look like as if they were measured in a near-saturation field.

> We relied on the automated functions of RockMagAnalyzer1.1 to analyse all our VFTB data. In the accompanying paper (Leonhardt, 2006), the author admits that, for non-saturated samples, the estimation of hysteretic parameters is not fully reliable, although it still allows the differentiation of low- and high-coercivity components in the samples: "Since saturation is not reached (...) hysteresis parameters are likely to be imprecisely estimated. Nevertheless, a low coercive (...) and a high coercive (...) component can be clearly identified after decomposition of the hysteresis loop. (Leondhardt, 2006: 1426); and "For interpretation of hysteresis parameters shown in (b) and (c) one has to keep in mind that saturation has not been reached in the experiment" (Leondhardt, 2006: 1427). Thus, our hysteretic parameters suffer from the same indetermination; we have added a note regarding this in lines 245-248. The previous stated field at which thermo-magnetic curves were carried out was, indeed, a mistake; thanks for noticing. We added the configuration file used for the VFTB in Table A in S1 Appendix, where you can see the pre-applied field is "30A"

(the standard units used by the equipment, corresponding to 1T). We have since changed the formulation in the main manuscript to *"a thermo-magnetic curve with a pre-applied field of 1T, heating up to 600°C and cooling back to room temperature"*.

2) Rock magnetism.
Samples used in this study are in most cases magnetically soft, with the exception of those like Q09.02r. At the same time, it seems an overly strong statement to refer to the coercive force ranging from 13 mT to 44 mT as "very low" (line 228). For a magnetite/maghemite-like phase, 'very low' would be something like <3-4 mT. Note that such samples would be dominated by multidomain grains and thus unsuitable for Thellier paleointensity experiments.

We changed the wording used to describe the coercivities of our materials. We now refer to them only as *"The hysteresis loops exhibit, generally, low coercivities and closed loops, with their coercive fields (Bc) ranging widely from 13 mT to 44 mT"* — acknowledging the variability while still emphasising that the materials are, for the most part, magnetically soft.

Considering the magnetic mineralogy, a conventional interpretation of a magnetically soft phase as low-Ti substituted or even pure magnetite (line 238) is probably not entirely correct. In archaeological ceramics sensu lato, magnetic phase(s) in most cases form during baking at high temperature, rather than inherited from a parent material. Ti is not the most likely element to substitute iron in the magnetite lattice during such a process; Al is equally if not more possible. In addition, vacancies could arise as well forming a phase intermediate between magnetite and maghemite [cf. Pilipenko, O. V., et al. (2019), Archaeomagnetic studies of the material of the archaeological monument Dmitrievskaya Sloboda II of the second millennium B.C, in Recent Advances in Rock Magnetism, Environmental Magnetism and Paleomagnetism, D. Nurgaliev, V. Shcherbakov, A. Kosterov and S. Spassov (Eds.), pp. 97-107, Springer International Publishing, Cham]. On the other hand, (near)-stoichiometric magnetite is not typical for ceramics, as attested by absence, in most cases, of the low-temperature Verwey phase transition [Kosterov, A., et al. (2021), High-coercivity magnetic minerals in archaeological baked clay and bricks, Geophys. J. Int., 224(2), 1256-1271; Troyano, M., et al. (2021), Analyzing the geomagnetic axial dipole field moment over the historical period from new archeointensity results at Bukhara (Uzbekistan, Central Asia), Phys. Earth Planet. Inter., 310, 106633].

We opened up our discussion regarding the magnetic carrier, including your remarks about the possibility of it being an aluminum-substituted phase instead of a Ti-magnetite, including the reference you suggested. You can now find these changes in lines 283-291. Unfortunately, we did not have the proper equipment to run low-temperature experiments, so the existence or absence of the Verwey transition in our samples could not be ascertained; the claim that the samples may contain almost-pure magnetite was based solely on Curie temperatures.

What do the authors mean under "a reversible peak between 100 and 200°C (Figure 3c)" (lines 249-250)? I rather see a Ms(T) curve that is slightly irreversible below 200°C. Note

that a similar but even stronger irreversibility is observed for the specimen shown in Figure 3d, that apparently contain an ε-$Fe2O3$-like phase in the initial state. During the Ms(T) run, extra epsilon-phase apparently forms, as attested by cooling curve going noticeably higher than the heating one. Similar but less pronounced process could explain the irreversibility of Ms(T) curve for the specimen Q22.03r (Figure 3c) as well.

> Regarding the behaviour of the cooling curves of some of the samples (Figure 3b), which display a slight mound in magnetisation between 100°C and 200°C and which we initially considered virtually reversible, we have accepted your suggestion, wording it in a very similar manner. You can now find this in lines 280-281.

It is mentioned (lines 201-202) that susceptibility vs. temperature curves were measured for selected samples. Please show a few examples in the SI section. Finally, it would be helpful if hysteresis parameters and Curie temperatures for all measured specimens were presented as a table in the Supporting Information. Include also the specimens' group according to color classification.

> We added magnetic susceptibility vs. temperature curves in the Supporting Information (Figure B in S1 Appendix) alongside the hysteretic parameters and Curie temperatures of all measured specimens (Table C in S1 Appendix). An extended table with Mrs/Ms and Bcr/Bc ratios is also provided separately in a spreadsheet on the associated repository.

3) Archaeomagnetic directions and intensities.
Authors accept as final and use for dating the directional results uncorrected for anisotropy whereas the intensities are corrected. This seems an inconsistent approach. It would be more straightforward to carry out dating using both uncorrected and corrected direction/intensity data sets.

> As you point out, initially we opted to preserve the uncorrected directions as final, which showed slightly better (smaller) $\alpha95$ values. However, since the differences between uncorrected and corrected directional values are not significant, we have accepted your suggestion —it is, indeed, more consistent to favour the corrected values for all parameters—. Table 2 in the main manuscript reflects now these changes. However, since TRM anisotropy and cooling rate corrections are of great importance to comply with high-quality standards, we did not think appropriate to perform a dating with the uncorrected values. The final dating is, thus, done with the corrected intensities and directions, and the new results displayed in Table 3 in the main manuscript.

Inspection of archaeointensity at the specimen level (Table SI1) shows that there are samples that require very large anisotropy correction, e.g. 07903B1 from kiln QLL07 and 9191 from QLL09. Resulting 'corrected' intensities deviate significantly from site means and are therefore suspect. Perhaps, it would be more prudent to reject intensities obtained from specimens requiring more than 10 % anisotropy correction.

Thanks for noticing samples with high TRMani corrections. We have checked our results and opted to exclude those with a TRMani correction factor > 1.02, both for intensity and directional estimations. While this deviates from your suggested threshold value of 1, we approached the values on a specimen-to-specimen basis, maintaining those that seemed in line with the most strong results in order to not diminish significantly the pool of available data.

In Table SI2, correction factors should be listed as well.

TRMani correction factors for each specimen are included under the heading "fani: TRM anistropy correction factor" (Table D in S1 Appendix).

In Table SI3, entries showing final results for kiln QLL09 apparently contain multiple typos. Values for corrected declination and inclination appear interchanged, and α95s seem wrong.

Thanks for noticing the mistakes in the full archaeodirectional results table. We have since corrected them (now in Table F in S1 Appendix).

4) Archaeomagnetic dating.
The comparison of archaeomagnetic directions and intensities is only made to global geomagnetic models (Figure 5). The problem is that these models use very few data from South America as an input. Perhaps it would be instructive to compare the intensity data obtained in this study also with the most recent South American reference curve [Goguitchaichvili, A., et al. (2023), Evolution of the Earth's magnetic field strength in northwestern Argentina during the last two millennia: Towards the improvement of south American geomagnetic paleosecular variation curve, J. South Amer. Earth Sci., 126, 104357].

We are in full agreement with your remark about global models smoothing out the characteristics of the field due to their scarcity of Southern Hemisphere data. This is a problem that we have noticed and also argued in our most recent work. However, the very low intensity values registered by the kilns, around 30μT, make impossible that they acquired their magnetisation in historical (meaning: post year 1 of the common era) moments previous to the modern configuration of the field and the presence of the South Atlantic Anomaly over South America. Thus, since we suspected a very recent re-magnetisation, we needed to compare our values against models which included similarly recent data, and especially those which included instrumental data. As such, local curves, such as those in Goguitchaichvili et al. (2023) or Gómez-Paccard et al. (2023), did not fit our purpose due to their lack of modern data.

5) Paper structure and presentation.
Text in lines 320 to 395 would be more appropriately placed in a separate section which may be provisionally entitled 'Archaeomagnetic dating'. The Discussion section would then start at line 397.

We created a new subsection for the archaeomagnetic dating of the kilns, which is now within the DISCUSSION attending also to the editor's suggestions. This is now in lines 392 and following of the reviewed manuscript.

Some figures are of inferior quality. In particular, Figure 3 is almost illegible. Please consider using different color scheme(s) to improve legibility.

We increased the resolution of all our images to 600dpi, and amended Figure 3 to improve legibility, as you suggested. The colours are now more contrasting and we have significantly reduced visual noise.

ANSWER TO REVIEWER 3

Dear reviewer,

Thanks for the time invested in our manuscript. We will address your concerns below, giving a detailed answer for each of the points you raised:

In my view, the work presented in this manuscript remains preliminary and incomplete. Throughout the manuscript, I found myself wondering why the authors sampled only the upper part of the combustion structure. The authors themselves acknowledge this limitation (line 451 and following). However, sampling the combustion chamber and the combustion floor should not be considered “beyond the scope of this paper,” as stated in line 465. Such sampling would have allowed a more informed discussion of the two hypotheses proposed to explain the archaeomagnetic results.

> As you mentioned, the sampling of both chambers of the kilns would have been ideal to cross-check if both chambers of the kilns had been re-heated. However, ideal conditions cannot always be attained. Archaeological work is required to comply with high-standards of documentation during field work, recording the totality of the findings and the excavation process. This means that, if we were to sample the lower chambers, a complete archaeological intervention would have been needed first. While we are not opposed to carry out this work —quite the contrary—, we could not foresee at the time of the sampling the results of our study: initially, we intended to perform an archaeomagnetic analysis in order to retrieve new information about the geomagnetic field in the past. We had no archaeological evidences that lead us to suspect a re-heating of the structures after their abandonement. Thusly, this was the rationale for sampling only those parts of the kilns that were already exposed, i.e. their upper chambers, a process that would effect the least possible damage on the un-excavated structures. We have since changed the wording in the main manuscript so as to reflect this circumstance, instead of simply stating that it is not the scope of the paper; you can now see the changes in a dedicated section in the DISCUSSION (lines 533-549).

In addition, a brief discussion of the radiocarbon (^{14}C) ages appears necessary. Why do these ages agree with the archaeological chronology, and why do they differ so markedly from the archaeomagnetic age? Furthermore, could the presence of significant amounts of magnetic metals near the furnaces have locally distorted the Earth’s magnetic field? To my knowledge, no experimental archaeomagnetic study has demonstrated the feasibility of reliable archaeomagnetic dating of metallurgical furnaces processing iron ore. Although the furnace studied here was used for copper extraction, it remains unclear whether iron ore was entirely absent.

> We have included a brief discussion about the available radiocarbon dates in the corresponding section (lines 147-166). We also explicitly acknowledged the discrepancy between C14 and archaeomagnetic dates in the DISCUSSION, providing a

plausible explanation for that, i.e. the fact that the charcoals used for C14 dating belong to Inka archaeological layers (lines 436-446). To our understanding, iron ore processing facilities would be perfectly suitable for archaeomagnetism, provided there were no cross-contamination of pure iron. However, this is not the case at Quillay, where no iron was found. Note that in the Inka and Colonial Andes copper and silver were the main metals (see, e.g., Lechtman, 2014); ironwork requires much higher temperatures, which in turn require different (industrial) structures. In addition, the type of pirquineo referred to in our work is not an smelting activity *per se* focused on the production of a final, refined metal; rather, it is a mining or recycling activity that would not have introduced extraneous ores within the kilns.

A complete sampling of the combustion structures would have reduced these uncertainties, including those related to the possible metallurgical function of the furnaces (particularly if the archaeomagnetic age had coincided with the ^{14}C age). In archaeological contexts, it is relatively common for both ^{14}C and archaeomagnetic ages to be younger than the archaeological age due to later reuse of structures. However, large discrepancies between ^{14}C and archaeomagnetic ages are uncommon. This discrepancy is what makes the present study potentially unique, and it is unfortunate that the underlying cause cannot be more precisely identified.

It is indeed unfortunate that we cannot fully ascertain the causes of the re-heating, but the current scientific evidence doesn't allow us to favour one hypothesis over the other, and we have expressed it as such in the main text (lines 581-591). This study has begotten a great deal of curiosity on our part as archaeologists and archaeomagnetists, demonstrating, in our opinion, the surprising turns that inter-disciplinary research can take. We hope to expand this work in the future in order to add more information on the patterns of use and abandonment at Quillay.

We now provide answers to your numbered comments for ease of reading:

1/ Title and throughout the manuscript: The manuscript uses the spelling "Inka" when referring to the Inca civilization. While "Inca" is the conventional spelling in English academic literature, "Inka" is sometimes used to reflect Quechua-based transliterations or specific cultural perspectives. If the use of "Inka" is intentional, this choice should be briefly justified and applied consistently throughout the manuscript. Otherwise, for clarity and consistency, the use of "Inca" is recommended.

Our choice of spelling Inka as it appears aligns with the Quechua spelling, rather than with the Spanish one. This is consistent with the accepted usage in many academic publications, especially those recently produced in South America (e.g. Iucci et al, 2024; González Godoy et al., 2025; or the volume edited by Hayashida, Troncoso & Salazar, 2022). Given that our manuscript is written in English, we see no benefit in using the Spanish orthography over the Quechua.

2/ Title : The title may need to be revised, as this hypothesis is not clearly supported by the results presented in this study.

> The title has been changed to exclusively reflect the empirical archaeomagnetic findings, i.e. the fact that the kilns were re-heated, without pointing to any particular hypothesis.

3/ Line 156: The orientation procedure of the plaster caps is unclear. Were sun sightings performed, or was only the magnetic azimuth measured? In the latter case, it is essential to explain how the magnetic declination correction was applied. In archaeomagnetism, high precision in sample orientation is critical, as even small orientation errors can result in significant temporal uncertainties when remanent magnetization directions are compared with secular variation reference curves.

> The orientation of the samples in the field was done with a sun compass, thus making corrections for magnetic declination unnecessary. The first version of the manuscript did not include this information; you can now find it in line 177.

4/ Line 160: Standard archaeomagnetic/paleomagnetic samples typically have a volume of approximately 8 cm^3, not 2 cm^3. Is this a typographical error, or were mini-samples used?

> The volume of our samples is, indeed, 8cm^3; the previous version was a mistake. Thanks for noticing; it is now changed as you can see in line 182.

5/ Line 182 and Supporting Information table: Reference #36 is incorrect, as it refers to a second paper by Gómez-Paccard (PEPI, 2006), which should be cited instead. The cooling-rate correction is grounded in physical principles (e.g., Néel theory) and is justified by the fact that, for single-domain grains, thermoremanent magnetization increases with slower cooling rates. Consequently, this correction necessarily reduces the estimated archaeointensity. When $F_{cr} > 1.0$, the correction should therefore not be applied. Although this error has appeared in several published studies, it should not be perpetuated unless theoretical justification is provided showing that cooling-rate correction can increase archaeointensity estimates. In archaeomagnetism, the formulation proposed by Veitch (1984) has often been used; this formulation differs from that adopted here, as Veitch divides the intensity by the correction factor rather than multiplying it. Additional details and clarification regarding the cooling-rate correction are therefore required.

> We changed the reference about the cooling rate correction to the correct one, Gómez-Paccard et al. (2006); thanks for noticing the mistake. We chose to apply cooling rate correction factors up to 1.02, thus increasing slightly the recommended maximum of $F_{cr} = 1$; which led us to eliminate some of the specimens and to the recalculation of intensities and directions. Our rationale for this was to implement your suggestion while retaining a significant number of specimens.

6/ Line 295: The sentence “Directional means were calculated using Fisher statistics” is awkward and potentially misleading. The mean direction obtained from the vector sum is independent of any distributional assumption, whereas the Fisher distribution applies only to the estimation of confidence parameters (e.g., κ, $\alpha95$). For clarity and statistical rigor,

these two steps should be explicitly distinguished (e.g., “the mean direction was calculated using the vector sum, and statistical parameters were computed assuming a Fisher distribution”), as stated in the reference cited by the authors.

> Thanks for noticing the inaccuracy in the wording regarding Fisher statistics. We have accepted your suggestion and changed the main text (lines 335-337); in addition, there is now more information about the experimental and analytical protocol in the Supplementary Information (renamed as S1 Appendix).

7/ Line 298: It is unclear how anisotropy correction could modify the MAD. In general, anisotropy correction is applied to the ChRM direction and does not affect the MAD. Even if the correction were applied to individual vectors during demagnetization, it is difficult to see how the MAD could change. This point requires clarification.

> The language about the effect of TRM anisotropy on the MAD value for directions was unclear, as you pointed. We have since changed our approach by the indication of another reviewer, including only values corrected for the TRM anisotropy effects, and thus this wording is no longer in the manuscript.

8/ Line 306: Please add confidence cones.

> You suggested adding “confidence cones” in line 306. We are unsure about which sentence this refers to; but since this section of the original manuscript referred to the uncorrected directional values and they have now been changed to the corrected directions, we believe this is no longer needed, as the text has disappeared. The α95 are included in Table 2, and have been added also in the main text (lines 352-353).

9/ Line 312: It is not the mean directions themselves that overlap, but rather their 95% confidence cones. Statistical tests would be required to support this interpretation.

> Changed “overlapping directions” for “overlapping α95” (line 362), thanks for the clarification.

10/ Line 313 (Figure 5a): This representation is not a stereographic projection. The plotting method is unconventional and potentially misleading. In equal-area or equal-angle stereographic projections, the y-axis does not correspond to declination. For example, a direction with I = 0 and D = 45 would lie outside the diagram shown. In addition, by convention in archaeomagnetism/paleomagnetism, negative inclinations are represented by open symbols rather than filled symbols.

> We have changed the caption in Figure 6 in order to reduce confusion. We have also changed the solid circles for open symbols in 5a, thanks for the suggestion.

Line 322: Replace “circles of confidence” with “confidence cones.”

> Changed “circles of confidence” for “confidence cones” (lines 379-380).

—

Sincerely,

Judit del Río (on behalf of all authors).
