# Supplementary material for: Archaeomagnetic evidence indicates post-Inka reheating of metallurgical kilns at Quillay (NW Argentina)
Source: PLoS One. 2026 May 27;21(5):e0350160. doi: 10.1371/journal.pone.0350160 (PMC13215555; doi:10.1371/journal.pone.0350160)
Supplement: Attachment — Submitted filename: letter_to_editor_reviewers.pdf [file pone.0350160.s004.pdf]

Madrid, May 5, 2026

Dear editor,

Please find a point-by-point answer to the issues you raised.

Kind regards,
Miriam Gómez-Paccard, on behalf of all authors.

The title, abstract, and keywords should be revised further for clarity and precision. The current title is much improved, but it remains somewhat heavy stylistically, and terms such as “shows” and “contemporary” still read as slightly stronger or less precise than necessary. A cleaner wording such as *Archaeomagnetic evidence indicates post-Inka reheating of metallurgical kilns at Quillay (NW Argentina)* may better reflect the evidential scope of the study.

Thanks for your suggestion, which seems appropriate to all authors and has been accepted.

The abstract is also improved, but several formulations still seem somewhat overstated in relation to the dataset, particularly “well-dated”, “strongly deviate”, and “clearly reveal”. The wording of the archaeomagnetic age estimate should also be aligned more closely with the date ranges discussed in the main text.

Thanks for your suggestion; we have adopted the abstract you proposed with some minor modifications to ease redundancies.

The keywords are generally relevant, but they should be made more specific and more consistent with the revised focus of the manuscript. One possible set would be: “archaeomagnetism; archaeomagnetic dating; archaeometallurgy; Andean archaeology; kiln reheating”.

We have accepted your suggestion and modified the keywords accordingly, thank you.

Second, please improve the presentation of Table 3. The table is informative, but its chronological message is not yet fully self-explanatory, particularly where some models yield disjoint age intervals within the 95% confidence range. Please clarify this more explicitly in the table layout, caption, or accompanying text, so that the reader can understand the meaning of these alternative intervals without having to infer the interpretation from the discussion alone.

Thanks you for your careful consideration on this repeated issue. While it is true that one of the global models (ArchKalMag) yielded two very different ages in some of the kilns, one of which is situated at the beginning of the Common Era, this only indicates that the values of the geomagnetic field are statistically compatible with those registered by the kilns. However, such an early dating was discarded on the basis of archaeological and historical evidence, as the industry and pottery retrieved at the site dates to much later. We have included clarifying text accordingly in lines 402-407 of the clean manuscript, and we have added an asterisk mark (*) and an explanation on the table caption in order to easily differentiate them from the valid intervals. We hope that this helps the readability of the information.

Third, please revise the evidential status of the argument supporting the re-smelting hypothesis. Part of this discussion currently relies on a statement attributed to “Pablo Cruz, personal communication”. This point should not remain in its current form. It should either be supported by citable and accessible evidence or reduced to a clearly marked ancillary observation that does not carry substantial interpretative weight.

We have erased the “personal communication” line, as it was part of our own team who conducted the analyses on site. We have indicated the preliminary nature of this assessment, centering the argument made by other scholars on the topic. You can find it in the clean copy of the manuscript, lines 518-523.

Finally, the manuscript requires a careful final language and copy-editing pass. There are still scattered typographical and editorial issues in the text, including missing spaces, minor spelling problems, and small punctuation inconsistencies. Please also ensure that the same cautious wording is maintained consistently across the title, abstract, Discussion, Conclusions, and figure captions.

We have unified the orthography of the manuscript to the United States norm, and we have conducted a line-by-line edit in order to get rid of ortho-typographical mistakes. Notwithstanding, some errors may remain. In addition, we adapted the wording used to express certainty in order to comply with your requests. We hope that this version of the manuscript better reflects the nature of our empirical study.
